# The Neuropharmacological Evaluation of Seaweed: A Potential Therapeutic Source

**DOI:** 10.3390/cells12222652

**Published:** 2023-11-18

**Authors:** Khoshnur Jannat, Rengasamy Balakrishnan, Jun-Hyuk Han, Ye-Ji Yu, Ga-Won Kim, Dong-Kug Choi

**Affiliations:** 1 Department of Applied Life Sciences, Graduate School, Konkuk University, Chungju 27478, Republic of Korea; jannat.koli.22@gmail.com (K.J.); digit0516@naver.com (J.-H.H.); oj7080@naver.com (Y.-J.Y.); gawon7425@naver.com (G.-W.K.); 2 Department of Biotechnology, Research Institute of Inflammatory Disease (RID), College of Biomedical and Health Science, Konkuk University, Chungju 27478, Republic of Korea; balakonkuk@kku.ac.kr

**Keywords:** neurodegenerative disease, aging, neuroinflammation, seaweed, bioactive compounds

## Abstract

The most common neurodegenerative diseases (NDDs), such as Alzheimer’s disease (AD) and Parkinson’s disease (PD), are the seventh leading cause of mortality and morbidity in developed countries. Clinical observations of NDD patients are characterized by a progressive loss of neurons in the brain along with memory decline. The common pathological hallmarks of NDDs include oxidative stress, the dysregulation of calcium, protein aggregation, a defective protein clearance system, mitochondrial dysfunction, neuroinflammation, neuronal apoptosis, and damage to cholinergic neurons. Therefore, managing this pathology requires screening drugs with different pathological targets, and suitable drugs for slowing the progression or prevention of NDDs remain to be discovered. Among the pharmacological strategies used to manage NDDs, natural drugs represent a promising therapeutic strategy. This review discusses the neuroprotective potential of seaweed and its bioactive compounds, and safety issues, which may provide several beneficial insights that warrant further investigation.

## 1. Introduction

Neurodegenerative disorders (NDDs), such as Alzheimer’s disease (AD) and Parkinson’s disease (PD), are the leading cause of mortality and morbidity among the elderly globally [1]. According to the World Health Organization, more than 35.6 million people suffer from dementia worldwide, with AD accounting for 60–70% of this population. The strongest risk factors for NDDs are oxidative stress, protein aggregation and misfolding, tau phosphorylation, neuroinflammation, and neuronal apoptosis, among other biological processes [2]. Other possible causes may include environmental risk factors, chemical exposure, immune and metabolic dysfunction, and genetic mutation associated with amyloid polymorphisms, mitochondrial mutations, and epigenetic changes, which can also be considered potential targets of neuroprotection [3,4].

The currently available NDD drugs provide only symptomatic relief, primarily by modulating neurotransmission instead of preventing the progression of the disease [5,6]. Recently, molecular target-based therapies have been introduced. These involve neurotransmitter modulators, direct receptor agonists and antagonists, second messenger modulators, stem cell-based therapies, neurotrophic factors, regulators of mRNA synthesis, and hormone replacement therapy to prevent disease-causing mutant proteins [7]. Two humanized monoclonal antibodies—lecanemab and donanemab—have been used to treat early AD. Lecanemab works selectively to eradicate the soluble amyloid protein, whereas donanemab functions against the N-truncated pyroglutamate amyloid beta (Aβ) peptide at position 3 (pGlu3-Aβ, AβpE3) through clearance facilitated by microglial cells [8,9]. Although these represent the only two currently available drugs for AD treatment, each can produce certain adverse effects. People with *APOE-ε4* gene mutations, particularly those who possess two copies of the mutation, might experience increased brain swelling (ARIA) in response to lecanemab treatment [10]. Donanemab has also produced some cases of ARIA and infusion-associated symptoms, including rash, dizziness, hot flashes, chills, and (in rare cases) cerebral microhemorrhage [11]. Numerous traditional symptomatic therapies lose their efficiency over time, yield disruptive symptoms of their own, and result in severe side effects [12]. Therefore, more effective and safer therapeutic drugs are urgently needed to prevent or slow the progression of NDDs.

A number of studies showed health-promoting properties in the use of natural products as potential therapeutics for neurodegeneration. Natural compounds have been reported to possess different biological activities, including antioxidant, anti-inflammatory, and antiapoptotic effects [13,14,15]. Because of a broad spectrum of pharmacological and biological activities, natural products are considered promising alternatives for the treatment or prevention of neurodegeneration as they might play a role in drug development and discovery [16,17,18,19]. Seaweed species, also known as macroalgae, are diverse and bountiful in the ocean. These photosynthetic eukaryotes are classified according to their pigmentation as Rhodophyta (or red), Chlorophyta (or green), or Phaeophyta (or brown seaweed). Seaweeds contain proteins, vitamins, minerals, and dietary fiber as well as important classes of bioactive compounds, such as polyphenols, polysaccharides, and sterols, which can have nutritional and medicinal qualities, such as antioxidant, anti-inflammatory, anticancer, and antidiabetes properties. Due to their many nutrients, seaweeds are a popular part of diets in some Asian countries, including Japan, China, Korea, and the Philippines. For example, in Japan, *Undaria pinnatifida* is known as wakame, *Hizikia fusiformis* is known as hijiki, *Laminaria* species are known as konbu, and red seaweed is known as nori [20,21,22]. Acknowledging that natural treatments have emerged as potential neuroprotective agents, this manuscript highlights the therapeutic potential of marine seaweeds and their bioactive compounds in the drive to mitigate NDD pathologies.

## 2. Pathophysiology of NDDs 

In patients with AD, the decrease in the number of hippocampal neurons in the brain and consequent cognitive decline ultimately contribute to the disease’s progression. The neuropathological characteristics of PD primarily include the accumulation of intracellular protein aggregates, Lewy bodies, and Lewy neuritis mainly due to the mistreated and aggregated forms of alpha-synuclein (α-synuclein) proteins and the gradual loss of nigrostriatal neurons [23]. Thus far, the pathogenesis of NDDs has not been fully explained. Moreover, patients with NDDs manifest an array of symptoms, including impairments to either or both cognitive memory and spatial learning, the inability to communicate, and motor issues, all of which are frequently associated with behavioral abnormalities [24]. Oxidative stress is a key component in the progression of NDDs. Excessive reactive oxygen species (ROS) production and poor antioxidant defenses cause oxidative stress that leads to brain damage [25]. Oxidative stress involves the excess production of ROS, which incurs damage to nucleic acids and small molecules, including proteins, DNA, and lipids. Oxidative stress can promote neuronal issues, specifically causing NDDs and accelerating cellular aging [26,27,28]. ROS (collectively, H_2_O_2_, OH, and O_2_^−^) may cause defects in mitochondrial respiration and the developmental processes of the human brain that are accompanied by augmented ROS generation. They also contribute to dynamic changes in the brain during neurodegeneration, and general aging [29,30,31]. As a primary source of energy production, mitochondria produce adenosine triphosphate (ATP) through oxidative phosphorylation (OXPHOS). The redox reaction that produces ATP is accomplished by the electron transport chain (ETC) complex (complexes I–V). Mitochondrial dysfunction can cause a deficit in the respiratory chain, increase ROS production, reduce ATP levels, trigger inflammation, and promote mitochondrial-dependent apoptosis, causing various NDDs [32,33].

Aging and most aging-related NDDs are related to impaired protein homeostasis. Protein aggregation, altered protein turnover, and post-translational modification are all hallmarks of NDDs [34]. In AD, the dual digestion of the amyloid precursor protein (APP) by the proteases β- and *γ*-secretase releases a subset of highly aggregative peptides, collectively termed Aβ, including Aβ_1–40_ and the highly aggregative Aβ_1–42_ [35,36]. Aβ aggregation results in neural loss, cognitive impairment, and eventually death. Similarly, the aggregation of α-synuclein underlies PD development [37]. Accumulating evidence suggests that tau oligomers (TauOs) prepared in vitro and derived from the brain tissue are the potent neurotoxic species causing synaptic dysfunction and impaired memory and are the seeds of intracellular tau aggregation in cellular models and in vivo [38,39,40]. Studies from human AD brains and animal models corroborate transcellular tau pathology propagation via anatomically connected brain regions, which is reminiscent of prion protein pathology [41,42]. Although the initiating factors of in vivo α-synuclein aggregation remain unknown, in vitro α-synuclein aggregation is influenced by chemical variabilities, such as pH and salt concentrations [43]. Furthermore, α-synuclein can cause nuclear and mitochondrial dysfunction, synaptic impairment, proteostasis imbalance, endoplasmic reticulum stress, and apoptosis [44].

**Figure 1:**
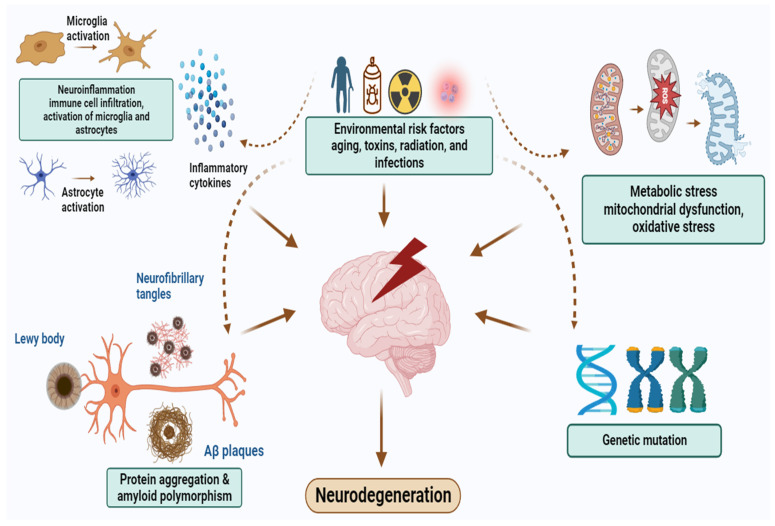
Hallmarks of neurodegenerative diseases and neuroprotective potential target of seaweeds. These include environmental risk factors, metabolic stress associated with mitochondrial dysfunction as well as oxidative stress, genetic contribution, misfolded protein aggregation, and neuroinflammation.

Increasing evidence has associated aging and NDDs with inflammation. Microglia are the primary immune cells in the central nervous system (CNS). They constitutively express surface receptors that trigger or increase the innate immune response, including chemokine receptors, cytokine receptors, complement receptors, complex II, and the major histocompatibility complex, all of which represent major neurotoxic factors in the progression of NDDs [45]. Activated microglia trigger the production of proinflammatory cytokines, including interleukin-1β (IL-1β), interleukin-6 (IL-6), and tumor necrosis factor alpha (TNF-α), as well as the production of prostaglandin E_2_ (PGE2), cyclooxygenase-2 (COX-2), nitric oxide (NO), and inducible nitric oxide synthase (iNOS), by activating mitogen-activated protein kinase (MAPK) (extracellular signal-regulated kinases (ERK)/c-Jun N-terminal kinases (JNK)), the protein kinase B (Akt) pathway, and the nuclear factor kappa B (NF-κB) pathway. Increased production of PGE2 by IL-1β causes synaptic loss, while TNF-α is responsible for neuronal death. Additionally, activated microglia exacerbate tau phosphorylation and tau pathology [46,47]. The abnormal accumulation of Aβ plaque causes the activation of the nucleotide-binding oligomerization domain (NOD)-like receptor protein 3 (NLRP3) inflammasome in microglia, which leads to caspase-1 activation, proinflammatory cytokine secretion, and eventually neuronal death [48]. Glial cells, such as astrocytes, are also responsible for neuroinflammation. One analysis of postmortem AD brain tissue demonstrated an accumulation of activated astrocytes around the Aβ deposits. Astrocytes also cause the production of proinflammatory cytokines, such as IL-1β and IL-6, resulting in neuronal loss [49]. Elsewhere, increased amounts of the proinflammatory cytokines IL-1β, IL-6, IL-2, TNF-α, and NO have been found in the substantia nigra of PD patients following brain analysis [50,51,52]. Figure 1 illustrates the hallmarks of neurodegeneration, as well as a neuroprotective potential target of seaweeds.

## 3. Pharmacology and Chemistry of Seaweed and Their Bioactive Compounds

**Figure 2:**
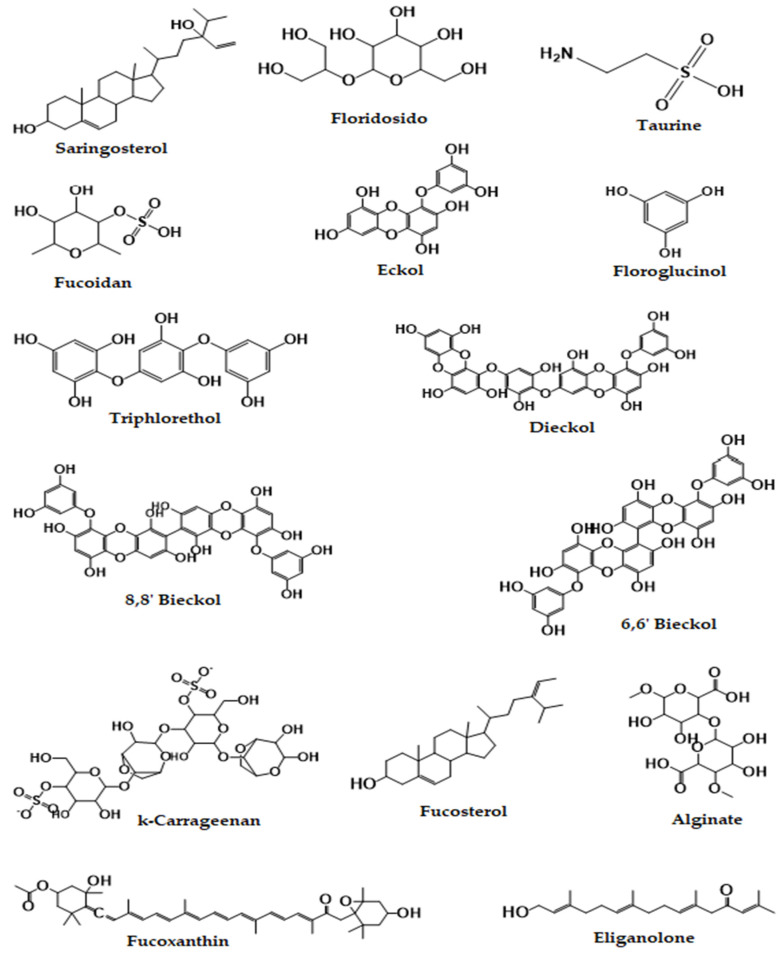
Structures of some chemical compounds of seaweeds.

Seaweed species are rich sources of a wide range of bioactive compounds known for their neuroprotective properties, such as polysaccharides, fatty acids, phlorotannin, carotenoids, conditionally essential amino acid (taurine), and phytosterol [53,54,55,56,57]. The chemical structure of seaweed bioactive compounds is described below (Figure 2). Seaweed extract and its bioactive compounds have been studied for their pharmacological properties. For instance, *H. fusiforme*, *Sargassum species*, and *Ulva lactuca* showed antioxidant, anti-inflammatory, neuroprotective, immunomodulatory, antitumor, anticancer, analgesic, antimicrobial, fibrinolytic, and antidiabetic effects [58,59,60]. 

Carrageenan, fucoidan, and alginate are the main polysaccharides found in seaweeds. Carrageenan can be categorized into seven fractions based on their recurring polysaccharide structure and the point of sulfate group attachment. The common use of carrageenan is gel formation; however, it was found to have antitumor, antiviral, and neuroprotective activities [61,62]. Alginate is a polymer of β-D-mannuronic acid and α-L-guluronic acid. This anionic polysaccharide and its derivatives exhibit a considered amount of solubility, thickening, biodegradability, pH sensibility, stabilizing, and gelling properties which make them excellent candidates for pharmaceutical applications mostly in the formation of nanocarrier formation [63]. Polymannuronic acid is one of the alginates that has shown anti-PD effects by reducing neuroinflammation [64]. Fucoidan is the most studied seaweed-derived fucose-rich sulfated polysaccharide. The chemical structure and bioactivity of fucoidan depend on the species they are isolated from; for example, fucoidan from *Fucus* species contains an extra acetate group [65]. Additionally, fucoidan isolated from *U. pinnatifida* is composed of fucose–galactose with a ratio of 1.1:1, and a small amount of uronic acid showed efficacy against inflammation with different mechanisms. This structure can increase IFN-γ (type II interferon), which is a cytokine that protects against inflammation [66]. IL-6 and TNF-α levels were attenuated by fucoidan with a ratio of Fuc:GlcA 6:1 with a high amount of uronic acid that was isolated from *Cladosiphon okamuranus* Tokida [67]. The plasma and tissue distribution of fucoidan was investigated by Pozharitskaya et al. 2018. A human equivalent dose of 100 mg/kg of fucoidan (735 kDa) was administered intragastrical in rats, and the plasma level was detected at a maximum concentration of 0.125 μg/mL at 4 h [68]. Through the parallel artificial membrane permeability assay (PAMPA), fucoidan, a seaweed polysaccharide, has demonstrated excellent BBB permeability [69].

Phlorotannins are composed of phloroglucinol monomers and exhibit a broad range of molecular sizes, ranging from 126 Da to 650 kDa. Phlorotannins display a wide range of bioactivity such as antimicrobial, antiallergic, anti-inflammatory, neuroprotective, and antioxidant properties. The bioactivity of phlorotannins also depends on their molecular masses; for example, 8–18 kDa of phlorotannins exhibits excellent antioxidant properties [70,71]. Eckol is one of the most important phlorotannins, which was found to have a 100% affinity to plasma protein, 55.60% intestinal absorption, and a moderate ability to penetrate the BBB [72]. Additionally, dieckol can cross the BBB to exert its neuroprotective effect [73].

Carotenoids consist of C40 hydrocarbon molecules in which isoprenoid works as building blocks, and from more than 750 carotenoids, 250 are derived from marine sources. Fucoxanthin (10%) is one of the most abundant carotenoids found in marine sources, and due to its allenic bond, it shows high antioxidant properties, which makes it a good neuroprotective candidate [74]. Fucoxanthin is a lipophilic pigment of brown seaweed that has also been observed to navigate the BBB when examined in vivo (200 mg/kg), indicating its ability to protect against neurological disorders [75]. 

Fucosterol is the main sterol compound of brown seaweed, with a formula of C_29_H_48_O. The amount of fucosterol produced by brown seaweed is higher than red and green seaweed. It was observed that dry leaves and stipes contain 312.0 μg/g and 378.1 μg/g fucosterol, which covers 98.6% and 98.9% of total sterol [76]. Wang et al. studied the pharmacokinetic parameters of fucosterol that was isolated from *S. fusiforme* in Sprague Dawley rats. They found that the plasma level of the compound was 0.300–18.0 μg/mL (bioavailability 0.74%), and it was excreted mainly through feces [77]. 

## 4. Neuroprotective Effects of Seaweeds and Their Bioactive Compounds in the Context of NDDs

This section discusses the therapeutic potential of seaweeds and their bioactive constituents to provide neuroprotective benefits to individuals with NDDs, such as AD and PD, and to protect against neuroinflammation and neuronal cell death. 

### 4.1. Neuroprotective Effects of Seaweed Extracts for NDDs

#### 4.1.1. In Vitro Studies

Seaweed extracts and their bioactive compounds have demonstrated various pharmacological effects, including neuroprotective activity and the promotion of neurite outgrowth [78]. For example, *Ecklonia radiata* (*E. cava*) is a brown seaweed popular in Australia. One study showed that three fractions, namely, polysaccharide, phlorotannin, and free sugar (100 µg/mL), effectively attenuated Aβ_1–42_-induced neuronal apoptosis and enhanced neurite outgrowth activity in PC-12 cells (pheochromocytoma) [79]. Similarly, Tirtawijaya et al. showed that the ethanolic extract of *K*. *alvarezii* effectively promotes the outgrowth of neurite and axonal development in hippocampal neurons [80,81]. Acetylcholinesterase (AChE) and butyrylcholinesterase (BChE) play important roles in NDDs such as AD. The use of AChE and BChE inhibitors in the treatment of AD has gained prominence due to the recognition that cholinergic deficiency represents the most consistent and earliest neurochemical anomaly observed in AD. Additionally, PD is characterized by dysregulation in the equilibrium of acetylcholine and dopamine neurotransmitters [82,83]. Inhibiting these two enzymes could be a therapeutic target for NDDs. The phlorotannin-rich extract of *E. cava* was also found effective for AChE and BChE inhibition and showed antioxidant activity against hydrogen peroxide (H_2_O_2_) and AAPH-induced oxidative damage in PC-12 and SH-SY5Y cells [84]. Meanwhile, a study concerning H_2_O_2_-induced neurotoxicity in PC-12 and MC-IXC cells showed that the fucoidan extract of *E. cava* could inhibit AChE, enhance antioxidant activity, and regulate mitochondrial function [85]. Seaweed including *Hypnea musiformis*, *Ochtodes secundiramea*, *Padina gymnospora*, *Codium tomentosum*, and *Pterocladiella capillacea* have various biological properties and have been reported to ameliorate or prevent Aβ_25–35_ aggregation and inhibit AChE and BuChE levels in different in vitro studies [86,87,88]. Another study reported that the aqueous ethanol extracts of *E. maxima* and *G. gracilis* exhibited significant AChE and BChE inhibitory activity and decumulation properties on Aβ_1–42_. *E. maxima* demonstrated radical scavenging activity at 82.8% according to an ABTS radical assay and above 60% according to a 2,2-diphenyl-1-picrylhydrazyl (DPPH) radical scavenging assay at a concentration of 3.09 mg/mL, in contrast to the results for *G. gracilis* [89]. The functional oil from *Hizikia fusiforme* showed AChE inhibitory activity, with an IC_50_ value of 1.00 ± 0.03 mg/mL, and 37% DPPH radical scavenging activity. Elsewhere, pretreatment with functional oil (20 mg/mL) significantly decreased NO and ROS production in LPS-stimulated BV-2 microglia cells [90].

The production of superoxide and H_2_O_2_ causes oxidative stress and subsequently damages the neuron cells [91]. The mitochondrial membrane potential (ΔΨm) plays a pivotal role in mitochondrial function, serving as a crucial element in ATP synthesis and electron transport, as well as in preserving mitochondrial structure, regulating apoptosis, maintaining calcium balance, and fostering the formation of new mitochondria. Ensuring its stability is paramount for the overall well-being and operation of the cell [92]. In a study regarding the neuroprotective effect of five seaweed species, namely, *Sargassum muticum* (*S. muticum*), *Saccorhiza polyschides* (*S. polyschides*), *Padina pavonica*, *Codium tomentosum*, and *Ulva compressa*, Silva et al. showed that the methanolic extracts of *S. muticum* and *S. polyschides* exhibited the highest neuroprotective effects against dopamine-induced neurotoxicity in SH-SY5Y cells. *S. muticum* and *S. polyschides* treatment significantly reduced H_2_O_2_ production, repolarized the ΔΨm, and decreased caspase-3-mediated cell death in SH-SY5Y cells. The authors performed another study with the same species to understand 6-hydroxydopamine (6-OHDA)-induced neurotoxicity on SH-SY5Y cells, and the extract demonstrated a positive result for neuroprotection [91,93]. Another in vitro study showed the neuroprotective effects of three fractions of fucoidan-rich extracts—namely, SH1, SH2, and SH3—isolated from *Sargassum hemiphyllum* against 6-OHDA-induced SH-SY5Y cells. Interestingly, treatment with all three extracts significantly improved cell viability, increased antioxidant activity, and considerably suppressed 6-OHDA-induced apoptosis in SH-SY5Y cells [94]. Elsewhere, the anti-AD effects of *Padina pavonica* (*P. pavonica*), a brown seaweed, on mitochondrial function and protein aggregation have been investigated in an Aβ-induced SH-SY5Y cell model, with the results showing that pretreatment with the acetone extract of *P. pavonica* could protect mitochondrial membrane potential (ΔΨm), inhibit α-synuclein and tau protein aggregation, and ameliorate the aggregation of the Aβ peptide formation in Aβ-induced SH-SY5Y cells [95].

However, the excessive production of NO activates NF-κB and induces the production of proinflammatory factors such as IL-1β, IL-6, PGE_2_, and TNF-α. The production of these proinflammatory factors in turn activates iNOS and COX-2 and further induces the body to produce more NO, thereby exerting a sustained toxic effect on cells [96]. Recent research has indicated that seaweeds, such as *Sargassum horneri* (S. horneri), have tremendous anti-neuroinflammatory and antioxidant activities. Their results showed that *S. horneri* extracts, the 70% EtOH extract (100 µg/mL), the CH_2_Cl_2_ soluble fraction (100 µg/mL), and the water-soluble fraction (200 µg/mL) significantly decreased the levels of PGE_2_, TNF-α, and IL-6 and reduced the iNOS and COX-2 expressions in LPS-induced BV-2 microglial cells. Moreover, *S. horneri* extracts prevented the activation of NF-κB/p-65 signaling in the nucleus, leading to increased heme oxygenase-1 (HO-1) expression in BV-2 microglial cells. Furthermore, the CH_2_Cl_2_ soluble fraction decreased glutamate-induced oxidative damage and ROS production in HT22 cells, suggesting that *S. horneri* has a potential anti-inflammatory effect [97]. Previous studies have demonstrated that SIRT1 is considered to be a vital modulator of cellular defenses and survival in response to stress [98,99,100]. It has also been found that impaired SIRT1 deacetylation induces oxidative stress [101] and that the overexpression of SIRT1 attenuates oxidative damage [102,103]. The extracts of *Undaria pinnatifida* (*U. pinnatifida*) and *Fucus vesciculosus* exhibited antioxidant and neuroprotective activity. The extracts also enhanced SIRT1 expression in MiaPaCa-2 cells and significantly reduced oxidative damage caused by iron in PC-12 cells [104,105]. 

#### 4.1.2. In Vivo Studies

In AD, the balance between Akt and GSK3-β activity is disrupted, leading to increased Aβ production, tau phosphorylation, neuronal dysfunction, and cell death. Researchers are actively investigating ways to modulate this pathway to potentially slow down or reverse the progression of AD [106]. An in vivo study was performed to compare the neuroprotective effects of fucoidan extract and polyphenol and a mixture (fucoidan extract–polyphenol = 4:6) of *E. cava* against a trimethyltin-induced AD in an ICR mice model. The results showed that the fucoidan extract and the 4:6 mixture improved spatial learning and memory function, decreased lipid peroxidation and AChE activity, restored the mitochondrial membrane potential and ATP content, and inhibited caspase-based apoptosis. Additionally, Akt/glycogen synthase kinase-3 beta (GSK-3β) expression was upregulated, and the expression of JNK/IRS and phosphorylated tau decreased, suggesting that the fucoidan extract and mixture (4:6) had greater cognitive effects than the polyphenol extract [107]. The neural tissue is highly sensitive to oxidative stress, and this is a prominent factor in both chronic and acute neurodegeneration. Even though the antioxidant defense systems like enzymatic antioxidants and non-enzymatic antioxidants are functioning, uncontrolled ROS accumulation during the life cycle promotes the development of age-dependent NDDs [108]. Reduced glutathione (GSH), an essential antioxidant, plays a pivotal role in preserving the well-being of cells, including those in the brain, by shielding them from the harmful effect of oxidative stress [109]. Um et al. investigated the anti-AD effects of a phlorotannin-rich fraction of *Ishige foliacea* (*I. foliacea*) extract (50 and 100 mg/kg). Their results showed that the phlorotannin-rich fraction of the *I. foliacea* extract significantly reduced malondialdehyde (MDA) levels and AChE activity, increased GSH and superoxide dismutase (SOD) activity, and improved spatial learning and cognitive function in a scopolamine-induced AD mouse model. In addition, the phlorotannin-rich fraction of the *I. foliacea* extract significantly elevated the scopolamine-induced downregulation of the ERK-cAMP response element-binding protein (CREB)–brain-derived neurotrophic factor (BDNF) signaling pathway [110].

Accumulating evidence has indicated that the upregulation of SIRT1 rescues neuronal loss, autophagy regulation, the promotion of neurogenesis, the maintenance of mitochondrial function, the modulation of energy metabolism, and anti-inflammatory action in acute and chronic NDDs [111]. The BDNF is a member of the neurotrophin family of growth factors. It plays a critical role in the growth, development, and maintenance of neurons, which are crucial for learning and memory [112]. More recently, *Ulva lactuca* (ULO) and *Enteromorpha prolifera* (EPO) treatment (150 mg/kg) ameliorated serum GLU, TG, and TC levels; reduced the levels of MDA; and led to significantly high levels of GSH, SOD, catalase, and T-AOC in the serum and brain tissue. In addition, ULO and EPO attenuated the increase in IFN-γ, TNF-α, IL-2, and IL-6 levels; increased the expression of SIRT1; and significantly elevated BDNF and ChAT levels in senescence-accelerated prone (SAMP8) mice [113]. Baek et al. investigated the anti-AD effect of the ethyl acetate extract from *Enteromorpha prolifera* on memory performance and neuronal apoptosis in an animal AD model, finding that the ethyl acetate extract (50 and 100 mg/kg) improved spatial learning and memory function. The underlying mechanism was partially associated with increased BDNF expression, the inhibition of Aβ and tau expression, and reduced AChE activity while subsequently attenuating neuronal damage from oxidative stress in the hippocampal CA1 and CA3 regions of ICR mice with scopolamine-induced AD [114]. Briffa et al. investigated the neuroprotective effects of acetone extracts isolated from the brown alga *Padina pavonica* (EPP) and the prickly pear *Opuntia ficus-indica* (EOFI), which alleviated neurodegenerative phenotypes in *Saccharomyces cerevisiae* and *Drosophila melanogaster* AD and PD models. Pretreatment with EPP and EOFI considerably enhanced the sustainability of yeast carrying the Arctic Aβ_42_ (E22G) mutant and substantially improved the survival and behavioral symptoms of flies with the brain-specific expression of wild-type Aβ_42_ (a model of late-onset AD) or the Arctic Aβ_42_ variant (a model of early-onset AD). Furthermore, EPP and EOFI extracts extended the lifespan of the PD fly, which was established through the transgenic expression of the human *α-syn A53T* mutant [115]. Table 1 summarizes the recent experimental findings on seaweeds and the effects of their bioactive constituents on NDDs.

### 4.2. Neuroprotective Effects of Bioactive Compounds in Seaweeds and Their Application in the Treatment of NDDs

Table 2 summarizes the recent experimental findings concerning the effects of these bioactive compounds on NDDs.

#### 4.2.1. Polysaccharides

The most common and commercially important polysaccharides found in seaweeds are alginate, agar, carrageenan, ulvan, and fucoidan [116]. These polysaccharides are isolated from the cell wall of seaweed species, for example, alginate from *Laminaria japonica* and *Ascophyllum*, carrageenan from *Hypnea*, and fucoidan from *Cladosiphon okamuranus* and *Ascophyllum nodosum*. These compounds and their derivatives exert neuroprotective effects against AD and PD [117,118,119].

In an in vitro model, alginate-derived oligosaccharide (500 µg/mL) was found to be effective against LPS-induced neuroinflammation and promoted the phagocytosis of Aβ. The results confirmed that pretreatment with alginate-derived oligosaccharide downregulated LPS- and Aβ-activated neuroinflammation by inhibiting protein and the mRNA expression levels of iNOS, COX-2, and PEG-2 and proinflammatory cytokines TNF-α, IL-6, and IL-1α, subsequently inhibiting the toll-like receptor 4 (TLR4)-NF-κB signaling pathway and decreasing the proinflammatory cytokine expression of IL-12 in BV-2 microglia cells [120]. In 6-hydroxydopamine (6-OHDA)-induced SH-SY5Y cells, pretreatment with kappa-carrageenan (κ-carrageenan) isolated from *Hypnea musciformis* (0.3 to 1.0 mg/mL) successfully reduced H_2_O_2_ production, improved mitochondrial function, and inhibited caspase-3 activity [121]. TLR4 activation can trigger the MAPK signaling pathway, leading to the activation of MAPKs such as p38 and JNK [122]. NF-κB is a key downstream target of TLR4 signaling. When TLR4 is activated by ligands such as LPS, it initiates a signaling cascade that ultimately leads to the activation of NF-κB [123,124]. NF-κB, once activated and translocated into the nucleus, promotes the expression of proinflammatory mediators, including cytokine and chemokines. Previous research findings have reported that drugs and other natural compounds alleviate disease symptoms mainly by inhibiting TLR4/NF-κB and p38/JNK MAPK signaling, microglia activation, and downstream proinflammatory cytokine production, thereby reducing oxidative stress and neuronal cell death and ultimately improving learning and cognitive function [125]. κ-Carrageenan oligosaccharides exhibited neuroprotective and anti-inflammatory effects on LPS-induced BV-2 microglia cells by significantly reducing NO activity and ROS and inhibiting the inflammatory response in the proinflammatory cytokines IL-1β, TNF-α, IL-6, and PGE2. κ-Carrageenan oligosaccharides have also been reported to act as a suppressor of the activation of TLR4/NF-κB and p38/JNK MAPK signaling pathways in LPS-induced BV-2 microglia cells [126]. Fucoidan, a major active component in brown seaweed species, has many pharmacological effects, including antioxidant and neuroprotective properties. In silico and in vitro studies have shown that fucoidan treatment can cross the BBB and reduce cytotoxicity, leading to an increase in the percentage of neurite length and the inhibition of AChE and BuChE activities. In an in vivo study, fucoidan treatment maintained the learning and memory function and significantly reduced the accumulation of protein and mRNA levels of Tau, PK13/AKT, and TNF-α and increased APP and beta-Secretase 1 (BACE-1) in the *Drosophila melanogaster* fly brain [69].

PGC-1α and NRF2 are two important proteins that play roles in cellular health, particularly in relation to oxidative stress, mitochondrial function, and neuroprotection. In the context of PD, both PGC-1α and NRF2 have garnered attention for their potential therapeutic significance [127,128]. Pretreatment with fucoidan (35, 70, and 140 mg/kg) dose-dependently protected against rotenone-induced behavioral abnormalities; reduced oxidative stress markers, including MDA, 3-NT, and 8-OHdG; and restored dopamine (DA) and its metabolites in the striatum of PD rats. In addition, fucoidan significantly reduced the loss of tyrosine hydroxylase (TH)-positive neurons in substantia nigra pars compacta (SNpc) and TH-positive fibers in the striatum, successfully restoring mitochondrial complex I and II activities and upregulating PGC-1α and nuclear factor erythroid 2-related factor 2 (NRF2) expressions in the ventral midbrain [129]. 

Ramu et al. demonstrated that treatment with fucoidan (100 and 200 mg/kg) significantly ameliorated behavioral deficits and addressed oxidative stress (reduced the MDA, AChE, and AGE contents and increased the GHS and SOD levels), the Aβ protein, and the hyperphosphorylation of the tau protein associated with streptozotocin-induced AD in rats. In addition, histochemical studies have demonstrated fucoidan to partially attenuate Aβ accumulation in the hippocampus and the cerebral cortex in streptozotocin-induced AD rats [130]. Polymannuronic acid, a natural alginate compound derived from brown seaweed, had a neuroprotective effect on an MPTP-induced PD animal model by improving motor function, preventing dopaminergic neuronal loss by increasing TH expression in the midbrain, enhancing serotonin (5-HT) and 5-hydroxyindole acetic acid (5-HIAA) levels in the striatum, and increasing the polymannuronic acid levels, which resulted in potential anti-inflammatory effects in PD mice [64].

#### 4.2.2. Fatty Acids

Fatty acids are abundant in various seaweeds, including *Asparagospis armata*, *Sargassum muticum*, *Gracilaria gracilis*, *Fucus vesciculosus*, *Ascophyllum nodosum*, *Saccharina latissimi*, *Bifurcaria bifurcate*, *S. horneri*, and *S. siliquastrum* [131,132,133,134,135]. The brain is highly enriched by fatty acids, including saturated fatty acids, monounsaturated fatty acids (MUFAs), and polyunsaturated fatty acids (PUFAs), which regulate both the structure and function of neurons, endothelial cells, and glial cells. In addition, many studies have revealed the crucial roles of fatty acids in neural synaptic plasticity, neuronal survival, the stimulation of neurogenesis and neurite outgrowth, and the regulation of brain neuroinflammation [136,137]. 

Studies have found that omega-3 PUFAs play key roles in the cell membrane structure and cytokine regulation and may be involved in regulating brain neuroinflammation [138,139]. Furthermore, decreased omega-3 PUFA levels have been shown to correlate with the onset of AD, suggesting that PUFA levels in the brain may be involved in cognition [140,141]. Dehkordi et al. investigated the effect of omega-3 PUFA on memory performance and neuroinflammation using an LPS-induced AD rat model. They found that PUFA treatment (400 mg/kg) significantly improved memory function and decreased cognitive deficiency. The underlying mechanism was partially associated with reduced TNF-α levels and increased calcium/calmodulin-dependent protein kinase type II subunit alpha (*CaMKII-α*) gene expression in the hippocampus in LPS-induced AD rats [142]. More recently, omega-3 PUFA treatment (1 g/kg) attenuated the increase in inflammatory cytokines IL-6 and TNF-α in the cerebral cortex of high-fat diet-induced rats [143]. Eicosapentaenoic acid (EPA) and docosahexaenoic acid (DHA), the major omega-3 PUFAs in fish oil, have demonstrated neuroprotective and anti-inflammatory properties. Pretreatment with EPA and DHA (200 µM) suppressed the production of the proinflammatory cytokines TNF-α and IL-6 and activated SIRT1 signaling by enhancing the mRNA level of nicotinamide phosphoribosyltransferase (NAMPT), SIRT1 protein deacetylase activity, cellular NAD^+^ levels, and *SIRT1* mRNA levels in LPS-stimulated MG6 and BV-2 microglia cells [144]. EPA and DHA effectively inhibited the phosphorylation of NF-κB activation and increased the autophagy indicators of the LC3-II/LC3-I ratio in LPS-stimulated MG6 cells and BV-2 microglia cells [145]. In an IL-1β-induced AD animal model, EPA treatment (25 and 30 mg/kg) reduced the biomarkers of microglial CD11b and astrocyte glial fibrillary acidic protein (GFAP) expression, decreased the expression of APP and TNF-α, and upregulated the BDNF and the expression of its receptor tyrosine receptor kinase B (TrKB) in the hippocampus. Furthermore, EPA supplements normalized the n-3 and n-6 PUFA profiles and cPLA2 levels, inhibiting neuroinflammation in the hippocampus in IL-1β-induced AD animals [146]. Dong et al. observed that the EPA (10 µM) showed the concentration-dependent neuroprotective effect of increased hippocampal neuronal viability, which significantly increased Akt/CREB expression and BDNF expression in conditions of IL-1β-induced neurotoxicity [147]. Similarly, Barroso-Hernández et al. reported that an omega-3 PUFA-rich oil supplement (300 mg/kg) had anti-PD effects, possibly mediated by the recovered locomotor activity and increased D_2_ receptor protein and gene expression in their haloperidol-induced PD rat model [148].

Taoro-González et al. found that an n-3 long-chain PUFA (n-3 LCPUFA) had neuroprotective and cognition-enhancing effects in a mouse model. Supplementation with n-3 LCPUFA (6.2, 7, 31, 40, and 50 mg/kg) improved spatial learning and object recognition memory and increased the subunits mGluR5 and N-methyl-D-aspartate (NMDA) GluN2B of glutamatergic receptors in aged C57BL/6 mice. Furthermore, n-3 LCPUFA significantly reduced the expression of proinflammatory cytokines, such as IL-1β and TNF-α, and reduced hippocampal microglia–astrocyte activation in aged C57BL/6 mice [149]. Similarly, a diet supplemented with 0.8% of n-3 PUFA–ethyl eicosapentaenoate (E-EPA) reversed impaired behavioral and motor functions; restored neurotransmitter contents; and suppressed proinflammatory cytokines, such as TNF-α, IFN-γ, and IL-10, in the cortex, hippocampus, and SNpc of MPTP-probenecid-induced PD mice [150]. Pretreatment with DHA (3 to 30 μM) inhibited IFN-γ-induced iNOS and COX-2 expression and NO production in IFN-α-induced BV-2 microglia cells. In addition, DHA increased HO-1 upregulation by modulating the PI3K/Akt and ERK signaling pathways, indicating antioxidant and anti-inflammatory activities [151]. Wu et al. found that DHA/EPA treatment (75 mg/kg) attenuated MPTP-induced deficits in motor coordination and behavioral function. The DHA/EPA diet promoted the neurodevelopment-related biomarker expressions of GAP-43 and BDNF, alleviated TH-positive neurons, significantly decreased the expression levels of p-JNK and p-P38, reduced oxidative damage, and downregulated proapoptotic protein expression (Bax and caspase-3) and antiapoptotic protein expression (Bcl2), subsequently downregulating the overexpression of p-GSK3β and p-Tau in the striatum of PD mice [152].

#### 4.2.3. Phlorotannins

Four phlorotannins (eckol, dieckol, 6,6′-bieckol, and 8,8′-bieckol) from the genus of brown seaweed *Ecklonia*, *Sargassum*, and *Fucus* have been reported to show antioxidant and neuroprotective effects [153]. 

Lee et al. observed that eckol, dieckol, and 8,8′-bieckol isolated from *Ecklonia cava* demonstrated potent neuroprotective activity against AD. Pretreatment with eckol, dieckol, and 8,8′-bieckol (1, 10, and 50 µM) exhibited neuroprotective effects in an Aβ_25–35_-induced PC-12 AD model. This was mediated by reduced cell death; decreased ROS generation; the induction of G0/G1-phase cell cycle arrest; the downregulation of the proapoptotic protein expression of cleaved caspase-8, -9, and -3, as well as PARP-1 and Bax; and the upregulation of the antiapoptotic protein expression of Bcl-2. In addition, the eckol, dieckol, and 8,8′-bieckol treatments reduced proinflammatory mediators (such as IL-1β, TNF-α, COX-2, and PGE-2) and inhibited the activation of MAPK and NF-κB signaling pathways in Aβ_25–35_-induced PC-12 cells [154]. It has been reported that phlorotannins such as eckol, dieckol, and 8,8′-bieckol exhibit potent inhibitory effects on BACE1 and AChE [155]. Meanwhile, another study reported that pretreatment with dieckol (1, 10, and 50 mM) exhibited the highest inhibitory effect on both intracellular and extracellular Aβ accumulation and regulated APP-processing enzymes, such as α-secretase (ADAM10), β-secretase, γ-secretase, and presenilin-1 (PS1), and their proteolytic products sAPPα and sAPPβ, and it significantly decreased Aβ_1–40_ and Aβ_1–42_ production and BACE-1 expression and increased ADAM-10 expression in APP^swe^ N2a cells. Furthermore, dieckol treatment successfully promoted the PI3K/Akt signaling pathway, which inactivated GSK-3β, resulting in reduced Aβ levels in SweAPP N2a cells [156].

#### 4.2.4. Carotenoids

Fucoxanthin, a carotenoid isolated from brown seaweed species, is effective against neuroinflammation, ROS, aging, and age-related NDDs [157,158]. 

In a study of murine BV-2 microglia cells, pretreatment with fucoxanthin (5, 10, and 50 µM) significantly reduced NO production and decreased the expression of the proteins iNOS, COX-2, and PGE2 and the proinflammatory cytokines IL-1β, IL-6, and TNF-α, subsequently inhibiting the MAPK signaling pathway in a concentration-dependent manner against Aβ_42_ neurotoxicity [159]. In glutamate-induced SH-SY5Y cells, fucoxanthin (8.25 µg/mL) caused attenuated neurotoxicity, successfully increased cell viability, and inhibited the activities of AChE and BuChE [160]. Similarly, Yu et al. demonstrated that fucoxanthin (0.3, 1, and 3 µM) significantly protects against H_2_O_2_-induced neuronal apoptosis and ROS. In addition, fucoxanthin increased the activity of the PI3K/Akt cascade, reduced the activity of the ERK pathway, significantly restored the altered activities of the PI3-K/Akt and ERK pathways, and protected H_2_O_2_-induced upregulated expression of the GSK3β and MAPK signaling pathways in SH-SY5Y cells [161]. Another research group reported that pretreatment with fucoxanthin (2 µM) mitigated the neurotoxicity of Aβ_1–42_ by considerably reducing Aβ_1–42_ fibrillation and increasing neurite outgrowth in PC-12 cells [162,163]. 

In Aβ_1–42_-induced SH-SY5Y cells, pretreatment with fucoxanthin at a dose of 0.1–1 µM considerably reduced Aβ_1–42_ fibril formation, decreased neuronal cytotoxicity, and elevated cell viability. In an in vivo model, fucoxanthin (100–200 mg/kg) improved recognition performance, spatial learning memory, and BDNF expression in Aβ_1–42_-treated AD mice [75]. A previous study demonstrated high-level expression of the DA3 receptor as an early sign of developing PD [164]. Fucoxanthin works as a DA3 agonist, as found in a study by Paudel et al. The EC_50_ of fucoxanthin for DA3 was 16.87. Human monoamine oxidase (hMAO) refers to two enzymes, MAO-A and MAO-B, that play crucial roles in the metabolism and breakdown of neurotransmitters in the brain including dopamine [165]. Considering its role in dopamine metabolism and its potential involvement in neurodegeneration, hMAO has been explored as a target for therapeutic interventions in PD. Fucoxanthin also exhibited human-MAO (hMAO) inhibitory activity with IC_50_ values of 197.41 and 211.12 for hMAO-A and hMAO-B, respectively [166]. In an MPTP-induced PD model, fucoxanthin (10 mg/kg) improved motor function, reduced the inflammatory responses mediated by decreasing COX-2 and iNOS protein expressions, and elevated the expression of the TH protein in C57BL/6 PD mice [167].

#### 4.2.5. Amino Acids

Seaweeds feature many health-promoting components, including vitamins, minerals, amino acids, dietary fibers, polysaccharides, and omega-3 and omega-6 fatty acids [168,169]. Taurine is a potent antioxidant and anti-inflammatory semi-essential amino acid extensively involved in NDDs, acting as a neurotrophic factor and blocking the excitotoxicity pathway, leading to a neuroprotective effect and neuromodulation [170].

Oh et al. reported that treatment with taurine (1000 mg/kg) significantly enhanced brain uptake of mGluR5, increased blood flow in the cerebra, and facilitated the recovery of the glutamate system in 5xFAD transgenic mice. However, they did not observe differences in Aβ pathology between the taurine-treated AD and AD groups in immunohistochemistry experiments [171]. In another study of Aβ oligomer-induced AD mice, taurine (250 mg/kg) treatment ameliorated spatial learning deficits and cognitive impairment by directly binding to oligomeric Aβ [172]. Treatment with taurine (200 mg/kg/day) improved manganese-induced spatial learning and cognitive functions, as determined by the enhanced ChAT activity and reduced AChE activity in a rat memory model [173]. Another study reported that taurine administration (1000 mg/kg) improved spatial learning and memory behavior and slightly decreased the insoluble fraction of Aβ in the hippocampus and the cortex in a transgenic APP/PS1 mouse model of AD [174]. SIRT1 is highly expressed in neurons and glial cells in the human brain and protects against neuronal damage by inhibiting oxidative stress and regulating mitochondrial function, which is strongly associated with NDDs [175]. An in vitro study by Terriente-Palacios et al. showed that taurine enhanced the activity of SIRT1 [176]. Several research groups have reported that taurine exerts neuroprotective and anti-inflammatory effects against paraquat-induced neurotoxicity via α-synuclein aggregation, modulating PI3K/Akt and MEK/ERK pathways and protecting dopaminergic neurons in PD mice [177,178,179,180]. Taurine combined with caffeine (8 mg/kg) enhanced rotational behavior and partially restored the DA levels in the striatum of rats with PD induced by 6-OHDA and apomorphine [181]. Taurine (150 mg/kg) treatment attenuated paraquat and the maneb-induced loss of TH-positive neurons in the noradrenergic locus coeruleus; inhibited microglial activation, M1 polarization, and the release of proinflammatory cytokines; and abrogated microglial NADPH oxidase activation and oxidative damage in PD mice. Furthermore, taurine inhibited the activation of NF-κB signaling but not signal transducers and activators of the STAT1/3 signaling pathway in PD mice [182].

#### 4.2.6. Sterols

In a study of APP^swe^/PS1^△E9^ mice, a 0.5 mg/25 g dose of 24(S)-saringosterol for 10 weeks prevented cognitive decline and significantly decreased the expression of the microglia activation marker Iba-1, but no synergetic effects were observed on Aβ plaque formation in the APP^swe^/PS1^△E9^ mice [183]. Liver X receptor β (LXRβ) is important for the motor neurons in the spinal cord and the maintenance of dopaminergic neurons in the SNpc, thus playing a role in the development of PD [184]. Notably, 24(S)-saringosterol isolated from *S. fusiforme* was found to be a novel selective agonist for LXRβ, indicating that 24(S)-saringosterol could be a potential leading compound for PD treatment [185]. Fucosterol, a phytosterol found in brown seaweed, exhibited neuroprotective effects on SH-SY5Y cells against Aβ-induced neurotoxicity. Fucosterol pretreatment (10 and 20 µM) reduced the mRNA levels of APP and upregulated the mRNA levels of neuroglobin (Ngb) in Aβ-induced SH-SY5Y cells, resulting in decreased intracellular Aβ levels [186]. Wong et al. reported that fucosterol had an anti-inflammatory effect on LPS-induced C8-B4 microglial cells: Pretreatment with fucosterol (12–192 µM) significantly inhibited NO production and the expression of proinflammatory cytokines, such as TNF-α, IL-1β, and IL-6, and successfully inhibited the LPS-induced increased AChE and BChE activity [187].

**Table 2:** Neuroprotective effects of the chemical compounds in seaweeds.

Class of Compound	Name of Compound	Model	Dose	Effect	References
Polysaccharide	Polymannuronic Alginate-derived oligosaccharide	Aβ and LPS-stimulated BV-2 microglia cells	500 µg/mL	Ameliorated neuroinflammation by inhibition of activation of the TLR4-NF-κB signaling pathway	[120]
	κ-Carrageenan	6-OHDA-induced SH-SY5Y cells and LPS-stimulated BV-2 microglia cells	0.3 to 1.0 mg/mL	Improved mitochondrial function, inhibited caspase-3 activity, and anti-inflammatory activity	[121,126]
	Fucoidan	PC-12 cells (treated with fucoidan for 7 days)	5–100 μg/mL	Enhanced neurite outgrowth	[69]
		Rotenone-induced PD rats	35, 70, and 140 mg/kg/b.w.	Reduced oxidative stress, enhanced dopamine content, and increased PGC-1α and NRF2 protein expressions	[129]
		Streptozotocin-induced AD rats	100 and 200 mg/kg/b.w.	Ameliorated behavioral deficits and reduced oxidative damage by increasing antioxidant enzyme activity	[130]
		MPTP-induced PD in male C57BL/6 mice	25 mg/kg/b.w.	Increased peripheral and central movement, reduced lipid peroxidation in the corpus striatum and midbrain, and increased TH and DAT protein expressions in the SNpc	[64]
Fatty acids	Omega-3 PUFA	LPS-induced AD in Rat	400 mg/kg/b.w.	Increased *CaMKII-α* gene expression and anti-inflammatory activity	[142]
		High-fat diet-induced rats	1 g/kg/b.w.	Reduced neuroinflammation by decreasing proinflammatory cytokines and inflammatory mediators	[143]
	Eicosapentaenoic acid and docosahexaenoic acid	LPS-stimulated MG6 cells and BV-2 microglia cells	200 µM	Reduced inflammation and increased *SIRT1* mRNA levels	[145]
		IL-1β-induced AD in rodent	25 and 30 mg/kg/b.w.	Upregulated BDNF and its receptor TrKB expression	[146]
		MPTP-induced PD in rat	75 mg/kg/b.w.	Elevated GAP-43 and BDNF expression and TH-positive neurons and downregulated overexpression of p-GSK3β and p-Tau	[152]
	Omega-3 PUFA-rich oil supplement	Haloperidol-induced PD rat	300 mg/kg/b.w.	Regained locomotor activity and alleviated the D_2_ receptor protein level	[148]
	n-3 long-chain PUFA	aged C57BL/6 mice	6.2, 7, 31, 40, and 50 mg/kg/b.w.	Improved spatial learning and object recognition memory and increased the NMDA subunits of mGluR5 and GluN2B of the glutamatergic receptors	[149]
	docosahexaenoic acid	IFN-α-induced BV-2 microglia cells	3 to 30 μM	Reduced neuroinflammation by regulating phosphorylation of PI3K/Akt and ERK signaling pathways	[151]
Phlorotannins	Eckol, dieckol, and 8,8′-bieckol	Aβ_25–35_-induced PC-12 cells	1, 10, and 50 µM	Antiapoptotic, antioxidative, and anti-neuroinflammatory properties	[154]
	Dieckol	APP^swe^ N2a cells and SweAPP N2a cells	1, 10, and 50 mM	Regulated the APP processing enzymes and the reduction of Aβ levels	[156]
Carotenoid	Fucoxanthin	Aβ_1–42_ treated BV-2 microglia cells	5, 10, and 50 µM	Anti-neuroinflammation	[159]
		Glutamate-induced SH-SY5Y cells	8.25 µg/mL	Inhibited AChE and BChE	[160]
		H_2_O_2_-induced SH-SY5Y cells	0.3, 1, and 3 µM	Upregulated expression of GSK3β, antiapoptotic, and anti-neuroinflammatory activities	[161]
		Aβ_1–42_-induced PC-12 cells	2 µM	Increased neural outgrowth	[162,163]
		Aβ_1–42_-treated AD mice	100–200 mg/kg/b.w.	Increased BDNF expression, spatial learning, and memory function	[75]
		MPTP-induced PD model	10 mg/kg/b.w.	Reduced neuroinflammation and improved motor function	[167]
Amino acid	Taurine	5xFAD transgenic mice	1000 mg/kg/b.w.	Enhanced brain uptake of mGluR5 and increased blood flow in the cerebra	[171]
		Aβ oligomer-induced AD mice	250 mg/kg/b.w.	Ameliorated special learning dysfunction and memory deficits	[172]
		MnCl_2_-treated Sprague–Dawley mice	200 mg/kg/b.w.	Enhanced the activity of ChAT and reduced AChE activity	[173]
		6-OHDA and apomorphine-induced PD rat	8 mg/kg/b.w.	Improved rotational behavior and partially replenished DA levels	[181]
		Paraquat and maneb-induced PD mouse	150 mg/kg/b.w.	Restored TH-positive neurons and inhibited activation of the STAT1/3 signaling pathway	[182]
Sterol	24(S)-saringosterol	APP^swe^/PS1^△E9^ mice	0.5 mg/kg/b.w.	Prevented cognitive decline and markedly reduced the expression of microglia activation	[183]
		Transfected HEK293T and HepG2 cells	0.5–40 µM	Selective agonist for LXRβ	[185]
	Fucosterol	Aβ-induced SH-SY5Y cells	10 and 20 µM	Increased levels of neuroglobin and reduced mRNA levels of APP	[186]
		LPS-induced C8-B4 microglial cells	12–192 µM	Decreased neuroinflammation and inhibited AChE and BChE activity	[187]

Aβ, amyloid beta; LPS, lipopolysaccharide; TLR4, Toll-like receptor 4; NF-κB, nuclear factor kappa B; 6-OHDA, 6-hydroxydopamine; PD, Parkinson’s disease; AD, Alzheimer’s disease; PGC-1α, peroxisome proliferator-activated receptor-gamma coactivator; Nrf2, nuclear factor erythroid 2-related factor 2; MPTP, 1-methyl-4phenyl-1,2,3,6-tetrahydropyridine; TH, tyrosine hydroxylase; DAT, dopamine transporter; CaMKII-α, calcium/calmodulin-dependent protein kinase type II subunit alpha; SNpc, substantial nigra pars compacta; SIRT1, sirtuin 1; mRNA, messenger RNA; BDNF, brain-derived neurotrophic factor; TrkB, tyrosine receptor kinase B; GAP-43, growth-associated protein; GSK3β, glycogen synthase kinase-3 beta; NMDA, N-methyl-D-aspartate; AKT, protein kinase B; ERK, extracellular signal-regulated kinases; PI3K, posphatidylinositol 3-kinases; AChE, acetylcholinesterase; BuChE, butylcholinesterase; APP, amyloid-beta precursor protein.

### 4.3. Toxicology of Seaweed and Its Bioactive Compounds 

Despite its beneficial compounds, seaweed also possesses some toxic elements that cause adverse effects on human health. These toxic effects are mostly due to heavy metals, with cadmium (Cd); lead (Pb); mercury (Hg); arsenic (As); and micronutrients, such as iodine (I), manganese (Mn), and nickel (Ni), all having been identified in considerable amounts in the species *Fucus*, *Ulva*, *Sargassum*, *Ascophyllum*, and *Saccharina* [188,189]. To investigate whether the consistent consumption of seaweed causes toxicity in humans, in vivo or in vitro studies have been performed using either seaweed extracts or their isolated heavy metals.

One recent study concerning the acute and subchronic oral toxicity of 90% ethanolic *Sargassum wightii* extracts and the effects on various rodent organs (corneal reflex, skin, fur, and eyes) revealed acute toxicity results, showing that the oral administration of 90% ethanolic *Sargassum wightii* extracts (2000 mg/kg) for 14 days did not cause any abnormalities in the animals. In addition, the subchronic experimental results showed that the oral administration of 90% ethanolic *Sargassum wightii* extracts (100, 200, and 400 mg/kg) for 28 days resulted in no significant change in the weight of the different organs or biochemical parameters, including albumin, creatinine, urea, cholesterol, ALT, and AST levels [190]. Similarly, Tapia-Martínez et al. observed that *Macrocystis pyrifera* powder mixed with commercial food is non-toxic up to 10 g kg^−1^. Acute and subchronic experiments revealed no signs of toxicity or changes in the hematological, biochemical, or histopathological parameters of the organs, indicating seaweed’s non-toxicity [191]. Taylor et al. studied the toxicity of commercially available seaweed species (nori, wakame, and kombu) using 11 volunteers. All participants were given 10 g of seaweed for three days. The urine analysis of the participants showed an increase in arsenosugars and the metabolites dimethyl arsenate (DMA), thio-dimethylarsinoylethanol (thio-DMAE), thio-dimethylarsinoylacetate (thio-DMAA), and thio-DMA, suggesting arsenic toxicity [192]. Various toxicity studies have shown that *Ecklonia cava* phlorotannins (at doses up to 400 mg/kg) have no adverse effects on hematological, clinical, or chemical parameters, except in one case characterized by nausea, dyspepsia, diarrhea, and alopecia [193,194,195]. 

Yun et al. performed a toxicity study with an enzymatic extract (300 g of pectinase and 200 g of cellulose) of *Ecklonia cava*. The study model used SD rats and beagle dogs for a single oral dose (3000 mg/kg), 14 days of repeated oral doses (1000, 2000, and 5000 mg/kg), and 13 weeks of repeated oral doses (500, 1000, 2000, and 3000 mg/kg). A dose of up to 3000 mg/kg resulted in a normal effect in the study model. The extract was also found to be non-clastogenic and non-mutagenic [196]. Fucoidan extracted from *Laminaria japonica* via enzymatic extraction was found to be safe at doses of 5000 mM/mL and 2000 mg/kg when studied in vitro and in vivo (SD rats, 28 days of administration). Because all biochemical parameters and organ features were normal, the use of the fucoidan supplement was found to be safe. Furthermore, a 2000 mg/kg dose of fucoidan did not exhibit any genotoxicity in the ICR mice [197,198]. Although there is no clinical trial for the toxicity studies of fucoxanthin, animal studies showed the safety measures of the compound. The Food and Drug Administration has permitted the consumption of fucoxanthin 3mg/day or 90 mg/day up to 90 days [199]. Fucosterol isolated from *S. fusiforme*, *U. pinnatifida*, and *H. fusiforme* with different concentrations did not show any toxic effects on different cell lines (3.125–500 μM) or in an animal study (50–100 mg/kg) [76]. Upon the request of the European Commission, the EFSA Panel on Dietetic Products, Nutrition, and Allergies tested the toxicity of phlorotannin isolated from *E. cava* for marketing the novel food supplement for individuals who are over 12 years old. This subchronic study was conducted by orally giving the dose 0, 375, 750, and 1500 mg/kg to rodents. The EFSA Panel found that it is safe for adolescents aged from 12 to 14 years and above 14 years to take phlorotannin food supplements at maximum doses of 163mg/day and 230 mg/day, respectively, while for adults, it is safe to consume 263 mg/day [200]. Dieckol is an important phlorotannin isolated from seaweed, which draws attraction for its diverse biological properties. There was no toxicity observed on human MRC-5 cells (human diploid cells) with a 400 μM concentration. The compound isolated from E. cava was found to be non-toxic to zebrafish with 50 μM. On the other hand, it showed mild side effects (diarrhea) on beagle dogs, with no mortality rate [201,202,203]. Although there are several toxicity studies of seaweed bioactive compounds, investigation with a proper experimental model is lacking and should be studied further. 

### 4.4. Recent Progressive Studies of Bioactive Compounds in Seaweeds

The marine-derived oligosaccharide sodium oligomannate recently received approval for AD treatment in China. Under the name of GV-971, the drug is available at a dose of 150 mg in an oral capsule, with a recommended dose of 450 mg twice a day. The drug was discovered by a team from the Ocean University of China, the Chinese Academy of Sciences Shanghai Institute of Material Medica, and Green Valley. A phase II trial was conducted with moderate AD patients (255 individuals) in two groups (600 mg and 900 mg) for 24 weeks. The phase II trial included 818 patients who received 900 mg of sodium oligomannate daily for 36 weeks. The drug works by inhibiting Aβ oligomerization and neuroinflammation. It can cross the BBB through the glucose transporter. The amount of Aβ _1-42_ in cerebrospinal fluid increased, indicating the degradation of Aβ_1–42_. There was also a reduction in the cerebral glucose metabolic rate in different regions of the brain. During the study, changes in the gut microbiota upon treatment were also observed [204,205,206,207]. Homotaurine or tramiprosate under the code name ALZ-801 was studied in a phase II trial on patients with mild AD (*APOE4/4* homozygotes). The patients were given 265 mg oral tablets twice daily, which resulted in the full inhibition of the formation of amyloid oligomer and showed a 40% penetration rate in the brain with no toxic effect. Currently, the drug is in a phase III trial with a dose of 530mg twice daily and is in the pipeline for FDA approval [208].

## 5. Current Limitations and Future Perspectives

There are several mechanisms involved in neurodegeneration that a single drug treatment can not cover. Therefore, multitargeted drug treatment strategies have been frequently proposed, where the compounds with multiple activities work on different mechanisms at different sites [209]. As discussed in Section 4, most of the seaweed compounds and extract revealed their neuroprotective effect by working on several mechanisms. Therefore, these compounds can be studied further for a single-dose compound or a food supplement, as they are derived from edible seaweeds and have shown no toxic effects, as previously discussed. Another main challenge for developing neurodegenerative drugs from seaweed compounds is the high rate of clinical trial failure and the lack of clinical studies. The majority of the studies stop at preclinical studies because of the lack of collaboration with researchers from other disciplines, for example, medical professionals and pharmaceuticals. Although there were several attempts at AD clinical trials, no data were found on clinical trials for PD treatment with seaweed-derived compounds. 

As seaweed contains a mixture of bioactive compounds, the isolation and purification of bioactive compounds at an industrial scale are challenging. For example, phenolic compounds (phlorotannin) are hard to isolate due to their structural similarity. The yield percentage of phlorotannin that is isolated from *S. fusiforme* is only 6.36, which is a very low amount for industrial purposes [210,211]. 

With bioavailability as an unavoidable factor, nanoformulations and encapsulations for drug delivery have now increased the percentage of bioavailability. Yasmin et al. reported that the nanoencapsulated polyphenol-rich extract of *Jania rubens*, a red seaweed, resulted in the successful sustained release of phytochemicals and showed antioxidant and neuroprotective properties [212]. Meanwhile, fucoidan has been reported to be involved in multiple biological activities. Owing to this diversity of impacts and its gel formation ability, fucoidan is currently used to prepare nanogels. Other seaweed polysaccharides, including chitosan, carrageenan, and alginate, are also popular candidates for the formation of nanogels [213,214]. Therefore, novel pharmaceutical delivery strategies may overcome the bioavailability issues of seaweed compounds, enhancing their potential effectiveness in humans. Further studies on the most effective delivery systems need to be performed, not only to validate their efficacy but also to address potential safety issues.

Due to the vast species and unique chemical composition, seaweeds provide a broad pool of compounds to explore for their neuroprotective potential. Also, seaweed cultivation is typically environmentally friendly and sustainable when compared to other sources of pharmaceutical compounds, such as synthetic chemicals or terrestrial plant extract. It has a minimal environmental impact. 

Preliminary results from pharmacokinetic studies indicate the rapid and significant brain permeability of seaweed compounds, which are expected to satisfactorily induce significant neuroprotective actions necessary to exert a beneficial therapeutic effect. However, further in vivo studies remain necessary to draw definitive conclusions regarding the ADME (absorption, distribution, metabolism, and excretion) properties and overall safety of seaweed compounds. Although there is a lack of clinical studies of seaweed-based bioactive compounds on neurodegenerative disease, sodium oligomannate is in the pipeline of being a successful drug for AD. Therefore, seaweed extract and its bioactive compounds have the potential to be studied as the leading compounds for neurodegenerative diseases such as AD and PD. 

## 6. Conclusions

**Figure 3:**
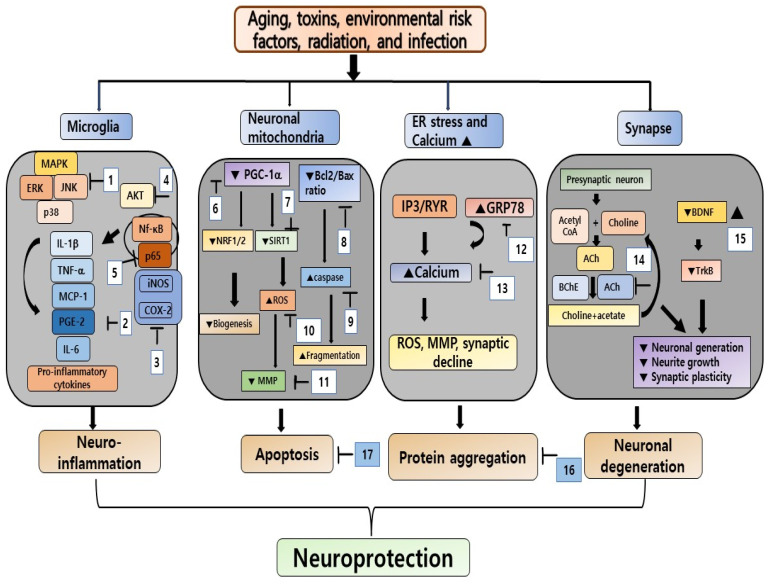
Pathophysiology of Alzheimer’s disease and Parkinson’s disease and the protective effect of seaweed extract and their bioactive compounds: 1. seaweed extracts, k-carrageenan, and fucoidan; 2, 3. seaweed extract, k-carrageenan, fucoidan, alginate, fucoxanthin, and taurine; 4. extract of *K. malesians* and *E. cava*, fucoidan, fucoxanthin, and carotenoid; 5. fucoidan; 6. fucoidan; 7. *U. lactuca* extract, taurine; 8. polymannuronic acid, fucoidan, phlorotannin, and fatty acids; 9. *Sargassum*, *E. cava*, *B. bifurcate*, *C. tomentosum* extract, and fucoidan; 10. seaweed extract, fucoidan, dieckol, fucoxanthin, and DHA; 11. *Sargassum*, *P. pavonica*, *B. bifurcata*, extract, dieckol, and eleganolone; 12. phycoerythrin; 13. phycoerythrin, dieckol; 14. *E. cava*, *P. gymnospora*, *E. prolifera*, *C. tomentosum*, *H. valentae* extracts, fucoidan, fucoxanthin, and phlorotannin; 15. *U. lactuca*, *I. foliacea*, *E. cava*, *E. prolifera* extracts, fucosterol, fucoxanthin, and fucoidan; 16. *E. prolifera*, *P. pavonica*, *S. fusiforme*, *A. nodosum*, *E. radiata*, *G. gracilis* extract, taurine, dieckol, fucoxanthin, and alginate; 17. sargassum extract, fucoxanthin, fatty acid, polymannuronic acid, k-carrageenan, fucoidan, and eleganolone.

This study provides a review of the literature on the use of marine algae for the treatment of NDDs. In addition to the typical widespread consumption of marine algae, cellular, animal, and other clinical experiments have reported the potential neuroprotective and antiaging effects of marine algae. In vitro and in vivo studies have focused on examining the anti-inflammatory and neuroprotective effects of extracts from marine algae (Figure 3). Although studies of marine algae have provided evidence of their potential neuroprotective effects, the specific underlying mechanisms have yet to be fully determined. Marine algae have been reported to have multiple bioactive metabolites and compounds with several pharmaceutical and biomedical applications. An investigation of the efficacy of the isolated bioactive compounds and their underlying neurobiochemical mechanisms of action is necessary before they are developed into novel anti-NDD agents. Notably, most of the clinical studies have been observational. Therefore, randomized controlled trials are still needed to verify the efficacy of marine algae in eliciting anti-inflammatory and neuroprotective effects before these products can be used clinically to treat individuals with NDDs.

## Figures and Tables

**Table 1:** A list of seaweed extracts and their neuroprotective effects on NDDs.

Name	Extraction Method	Model	Dose	Effect	References
*Ecklonia cava*	Polysaccharide, phlorotannin-rich extract	Aβ_1–42_-induced PC-12	100 µg/mL	Exhibited antiapoptotic and neurite outgrowth-enhancing properties	[79]
*K*. *alvarezii*	Ethanolic extract	Fetal rat hippocampal neuron	1 µg/mL	Promotes neural outgrowth	[80,81]
*Ecklonia cava*	Phlorotannin-rich extract	H_2_O_2_ and AAPH-induced PC-12, SH-SY5Y	62.5 and 30 µg/mL	Inhibition of AChE and BChE activities and antioxidant-enhancing properties	[82]
	Fucoidan-rich extract	H_2_O_2_-induced PC-12 and MC-IXC cells	50 and 100 µg/mL	Regulation of mitochondrial function	[83]
*E. maxima* and *G. gracilis*	Aqueous extract	In vitro assay (Aβ_1–42_ was incubated with extract for 0–96 h)	3.09 mg/mL	Inhibition of Aβ_1–42_ accumulation and antioxidant activity	[87]
*Hizikia fusiforme*	Functional oil	LPS-stimulated BV-2 microglia cells	1.00 ± 0.03 mg/mL, 20 mg/mL	Exhibited AChE and BChE inhibition, and antioxidant and anti-inflammation activities	[88]
*S. muticum* and *S. polyschides*	Methanolic extract	Dopamine and 6-OHDA-induced SH-SY5Y	1000 µg/mL	Repolarization of the mitochondrial membrane potential	[89,91]
*Sargassum hemiphyllum*	Fucoidan-rich extract	6-OHDA-induced SH-SY5Y	500 µg/mL	Exhibited antioxidant and antiapoptotic properties	[94]
*P. pavonica*	Acetone extract	Aβ-induced SH-SY5Y cell	50 µg/mL	Maintained mitochondrial function and inhibition of protein aggregation	[95]
*S. horneri*	70% EtOH extract, CH_2_Cl_2_ soluble fraction, and water-soluble fraction	LPS-induced BV-2 microglia cells	100 µg/mL, 100 µg/mL, and 200 µg/mL	Exhibited anti-inflammatory potential by preventing the activation of NF-κB/p-65 signaling	[97]
*U. pinnatifida*, *F. vesciculosus*	Whole-plant extract	MiaPaCa-2 cells (Human pancreatic epithelial cells pretreated with extract)	4.9 and 14.8 µg/mL	Enhanced SIRT1 expression and antioxidant activity	[104]
*U. pinnatifida*	Whole-plant extract	Iron-induced PC-12 cells	200 µg/mL	Reduces oxidative damage by reducing lipid peroxidation and restoring antioxidant enzyme activities	[105]
*E. cava*	Fucoidan and polyphenol extract	Trimethyltin-induced ICR mice	A mixture of fucoidan and polyphenol in a 4:6 ratio	Improves the spatial learning and memory function, restoration of mitochondrial membrane potential, AChE inhibition, and upregulation of Akt/GSK-3β expression	[107]
*I. foliacea*	Phlorotannin-rich extract	Scopolamine-induced AD mouse	50 and 100 mg/kg/b.w.	Improved spatial learning and cognitive function and upregulated ERK-CREB-BDNF signaling	[110]
*Ulva lactuca* and *Enteromorpha prolifera*	Aqueous extract	Senescence-accelerated prone (SAMP8) mice	150 mg/kg/b.w.	Exhibited anti-inflammatory activity, increased expression in Sirt1, and elevated BDNF and ChAT levels	[111]
*Enteromorpha prolifera*	Ethyl acetate extract	Scopolamine-induced AD ICR mice	50 and 100 mg/kg/b.w.	Improved spatial learning and memory function, increased BDNF expression, and inhibited Aβ and tau expression	[115]

Aβ, amyloid beta; H_2_O_2_, hydrogen peroxide; 6-OHDA, 6-hydroxydopamine; AAPH, 2,2′-Azobis(2-amidinopropane) dihydrochloride; AChE, acetylcholinesterase; BuChE, butyl cholinesterase; LPS, lipopolysaccharide; EtOH, ethanol; NF-κB, nuclear factor kappa B; SIRT1, sirtuin 1; AKT, protein kinase B; GSK3β, glycogen synthase kinase-3 beta; ERK, extracellular signal-regulated kinases; CREB, cAMP response element-binding protein; BDNF, brain-derived neurotrophic factor.

## Data Availability

Not applicable.

## References

[1] Wyss-Coray T. (2016). Ageing, Neurodegeneration and Brain Rejuvenation. Nature.

[2] Kovacs G.G., Budka H. (2010). Current Concepts of Neuropathological Diagnostics in Practice: Neurodegenerative Diseases. Clin. Neuropathol..

[3] Peden A.H., Ironside J.W. (2012). Molecular Pathology in Neurodegenerative Diseases. Curr. Drug Targets.

[4] Tycko R. (2015). Amyloid Polymorphism: Structural Basis and Neurobiological Relevance. Neuron.

[5] Breijyeh Z., Karaman R. (2020). Comprehensive Review on Alzheimer’s Disease: Cause and Treatment. Molecules.

[6] Mortada I., Farah R., Nabha S., Ojcius D.M., Fares Y., Almawi W.Y., Sadier N.S. (2021). Immunotherapies for Neurodegenrative Diseases. Front. Neurol..

[7] Balakrishnan R., Kim Y.-S., Kim G.-W., Kim W.-J., Hong S.-M., Kim C.-G., Choi D.-K. (2023). Standardized Extract of Glehnia Littoralis Abrogates Memory Impairment and Neuroinflammation by Regulation of CREB/BDNF and NF-κB/MAPK Signaling in Scopolamine-Induced Amnesic Mice Model. Biomed. Pharmacother..

[8] Van Dyck C.H., Swanson C.J., Aisen P., Bateman R.J., Chen C., Gee M., Kanekiyo M., Li D., Reyderman L., Cohen S. (2023). Lecanemab in Early Alzheimer’s Disease. N. Eng. J. Med..

[9] Lowe S.L., Willis B.A., Hawdon A., Natanegara F., Chua L., Foster J., Shcherbinin S., Ardayfio P., Sims J.R. (2021). Donanemab (LY3002813) Dose-escalation Study in Alzheimer’s Disease. Alzheimer’s Dement. Transl. Res. Clin. Interv..

[10] Qiao Y., Chi Y., Zhang Q., Ma Y. (2023). Safety and Efficacy of Lecanemab for Alzheimer’s Disease: A Systematic Review and Meta-Analysis of Randomized Clinical Trials. Front. Aging Neurosci..

[11] Rashad A., Rasool A., Shaheryar M., Sarfraz A., Sarfraz Z., Robles-Velasco K., Cherrez-Ojeda I. (2022). Donanemab for Alzheimer’s Disease: A Systematic Review of Clinical Trials. Healthcare.

[12] Balakrishnan R., Park J.-Y., Cho D.-Y., Ahn J.-Y., Yoo D.-S., Seol S.-H., Yoon S.-H., Choi D.-K. (2023). AD−1 Small Molecule Improves Learning and Memory Function in Scopolamine-Induced Amnesic Mice Model through Regulation of CREB/BDNF and NF-κB/MAPK Signaling Pathway. Antioxidants.

[13] Reuter S., Gupta S.C., Chaturvedi M.M., Aggarwal B.B. (2010). Oxidative Stress, Inflammation, and Cancer: How Are They Linked?. Free Radic. Biol. Med..

[14] Hald A., Lotharius J. (2005). Oxidative Stress and Inflammation in Parkinson’s Disease: Is There a Causal Link?. Exp. Neurol..

[15] Sánchez-López F., Tasset I., Agüera E., Feijóo M., Fernández-Bolaños R., Sánchez F.M., Ruiz M.C., Cruz A.H., Gascón F., Túnez I. (2012). Oxidative Stress and Inflammation Biomarkers in the Blood of Patients with Huntington’s Disease. Neurol. Res..

[16] Rojas-Gutierrez E., Muñoz-Arenas G., Treviño S., Espinosa B., Chavez R., Rojas K., Flores G., Díaz A., Guevara J. (2017). Alzheimer’s Disease and Metabolic Syndrome: A Link from Oxidative Stress and Inflammation to Neurodegeneration. Synapse.

[17] Lamptey R.N.L., Chaulagain B., Trivedi R., Gothwal A., Layek B., Singh J. (2022). A Review of the Common Neurodegenerative Disorders: Current Therapeutic Approaches and the Potential Role of Nanotherapeutics. Int. J. Mol. Sci..

[18] Atanasov A.G., Zotchev S.B., Dirsch V.M., Supuran C.T. (2021). Natural Products in Drug Discovery: Advances and Opportunities. Nat. Rev. Drug Discov..

[19] Oguntibeju O.O. (2019). Type 2 Diabetes Mellitus, Oxidative Stress and Inflammation: Examining the Links. Int. J. Physiol. Pathophysiol. Pharmacol..

[20] Murai U., Yamagishi K., Kishida R., Iso H. (2021). Impact of Seaweed Intake on Health. Eur. J. Clin. Nutr..

[21] Ren C., Liu Z., Wang X., Qin S. (2022). The Seaweed Holobiont: From Microecology to Biotechnological Applications. Microb. Biotechnol..

[22] Baghel R.S., Reddy C.R.K., Singh R.P. (2021). Seaweed-Based Cellulose: Applications, and Future Perspectives. Carbohydr. Polym..

[23] Kovacs G.G. (2014). Current Concepts of Neurodegenerative Diseases. EMJ Neurol..

[24] Balakrishnan R., Cho D.-Y., Kim I.-S., Seol S.-H., Choi D.-K. (2022). Molecular Mechanisms and Therapeutic Potential of α- and β-Asarone in the Treatment of Neurological Disorders. Antioxidants.

[25] Butterfield D.A., Halliwell B. (2019). Oxidative Stress, Dysfunctional Glucose Metabolism and Alzheimer Disease. Nat. Rev. Neurosci..

[26] Dalleau S., Baradat M., Guéraud F., Huc L. (2013). Cell Death and Diseases Related to Oxidative Stress:4-Hydroxynonenal (HNE) in the Balance. Cell Death Differ..

[27] Gella A., Durany N. (2009). Oxidative Stress in Alzheimer Disease. Cell Adhes. Migr..

[28] Cassidy L., Fernandez F., Johnson J.B., Naiker M., Owoola A.G., Broszczak D.A. (2020). Oxidative Stress in Alzheimer’s Disease: A Review on Emergent Natural Polyphenolic Therapeutics. Complement. Ther. Med..

[29] Bhat A.H., Dar K.B., Anees S., Zargar M.A., Masood A., Sofi M.A., Ganie S.A. (2015). Oxidative Stress, Mitochondrial Dysfunction and Neurodegenerative Diseases; a Mechanistic Insight. Biomed. Pharmacother..

[30] Guo C., Sun L., Chen X., Zhang D. (2013). Oxidative Stress, Mitochondrial Damage and Neurodegenerative Diseases. Neural Regen. Res..

[31] Kim T.Y., Leem E., Lee J.M., Kim S.R. (2020). Control of Reactive Oxygen Species for the Prevention of Parkinson’s Disease: The Possible Application of Flavonoids. Antioxidants.

[32] Bhatia V., Sharma S. (2021). Role of Mitochondrial Dysfunction, Oxidative Stress and Autophagy in Progression of Alzheimer’s Disease. J. Neurol. Sci..

[33] Bell S.M., Barnes K., De Marco M., Shaw P.J., Ferraiuolo L., Blackburn D.J., Venneri A., Mortiboys H. (2021). Mitochondrial Dysfunction in Alzheimer’s Disease: A Biomarker of the Future?. Biomedicines.

[34] Sengupta U., Kayed R. (2022). Amyloid β, Tau, and α-Synuclein Aggregates in the Pathogenesis, Prognosis, and Therapeutics for Neurodegenerative Diseases. Prog. Neurobiol..

[35] Pîrşcoveanu D.F.V., Pirici I., Tudorică V., Bălşeanu T.A., Albu V.C., Bondari S., Bumbea A.M., Pîrşcoveanu M. (2017). Tau Protein in Neurodegenerative Diseases—A Review. Rom. J. Morphol. Embryol..

[36] Ashrafian H., Zadeh E.H., Khan R.H. (2021). Review on Alzheimer’s Disease: Inhibition of Amyloid Beta and Tau Tangle Formation. Int. J. Biol. Macromol..

[37] Mehra S., Sahay S., Maji S.K. (2019). α-Synuclein Misfolding and Aggregation: Implications in Parkinson’s Disease Pathogenesis. Biochim. Biophys. Acta Proteins Proteom..

[38] Puzzo D., Piacentini R., Fá M., Gulisano W., Li Puma D.D., Staniszewski A., Zhang H., Tropea M.R., Cocco S., Palmeri A. (2017). LTP and Memory Impairment Caused by Extracellular Aβ and Tau Oligomers Is APP-Dependent. eLife.

[39] Wang Y., Balaji V., Kaniyappan S., Krüger L., Irsen S., Tepper K., Chandupatla R., Maetzler W., Schneider A., Mandelkow E. (2017). The Release and Trans-Synaptic Transmission of Tau via Exosomes. Mol. Neurodegener..

[40] Fá M., Puzzo D., Piacentini R., Staniszewski A., Zhang H., Baltrons M.A., Li Puma D.D., Chatterjee I., Li J., Saeed F. (2016). Extracellular Tau Oligomers Produce An Immediate Impairment of LTP and Memory. Sci. Rep..

[41] Vasili E., Dominguez-Meijide A., Outeiro T.F. (2019). Spreading of α-Synuclein and Tau: A Systematic Comparison of the Mechanisms Involved. Front. Mol. Neurosci..

[42] Vogel J.W., Iturria-Medina Y., Strandberg O.T., Smith R., Levitis E., Evans A.C., Hansson O., Weiner M., Aisen P., Petersen R. (2020). Spread of Pathological Tau Proteins through Communicating Neurons in Human Alzheimer’s Disease. Nat. Commun..

[43] Buell A.K., Galvagnion C., Gaspar R., Sparr E., Vendruscolo M., Knowles T.P.J., Linse S., Dobson C.M. (2014). Solution Conditions Determine the Relative Importance of Nucleation and Growth Processes in α-Synuclein Aggregation. Proc. Natl. Acad. Sci. USA.

[44] Du X., Xie X., Liu R. (2020). The Role of α-Synuclein Oligomers in Parkinson’s Disease. Int. J. Mol. Sci..

[45] Calsolaro V., Edison P. (2016). Neuroinflammation in Alzheimer’s Disease: Current Evidence and Future Directions. Alzheimer’s Dement..

[46] Leng F., Edison P. (2021). Neuroinflammation and Microglial Activation in Alzheimer Disease: Where Do We Go from Here?. Nat. Rev. Neurol..

[47] Kwon H.S., Koh S.-H. (2020). Neuroinflammation in Neurodegenerative Disorders: The Roles of Microglia and Astrocytes. Transl. Neurodegener..

[48] Zhang Y., Zhao Y., Zhang J., Yang G. (2020). Mechanisms of NLRP3 Inflammasome Activation: Its Role in the Treatment of Alzheimer’s Disease. Neurochem. Res..

[49] Liao Y., Xing Q., Li Q., Zhang J., Pan R., Yuan Z. (2021). Astrocytes in Depression and Alzheimer’s Disease. Front. Med..

[50] Gelders G., Baekelandt V., Van der Perren A. (2018). Linking Neuroinflammation and Neurodegeneration in Parkinson’s Disease. J. Immunol. Res..

[51] Badanjak K., Fixemer S., Smajić S., Skupin A., Grünewald A. (2021). The Contribution of Microglia to Neuroinflammation in Parkinson’s Disease. Int. J. Mol. Sci..

[52] Belarbi K., Cuvelier E., Bonte M.-A., Desplanque M., Gressier B., Devos D., Chartier-Harlin M.-C. (2020). Glycosphingolipids and Neuroinflammation in Parkinson’s Disease. Mol. Neurodegener..

[53] Barbosa M., Lopes G., Andrade P.B., Valentão P. (2019). Bioprospecting of Brown Seaweeds for Biotechnological Applications: Phlorotannin Actions in Inflammation and Allergy Network. Trends Food Sci. Technol..

[54] Schepers M., Martens N., Tiane A., Vanbrabant K., Liu H.-B., Lütjohann D., Mulder M., Vanmierlo T. (2020). Edible Seaweed-Derived Constituents: An Undisclosed Source of Neuroprotective Compounds. Neural Regen. Res..

[55] Patel S. (2018). Seaweed-Derived Sulfated Polysaccharides. Bioactive Seaweeds for Food Applications.

[56] Sohn S.-I., Rathinapriya P., Balaji S., Jaya Balan D., Swetha T.K., Durgadevi R., Alagulakshmi S., Singaraj P., Pandian S. (2021). Phytosterols in Seaweeds: An Overview on Biosynthesis to Biomedical Applications. Int. J. Mol. Sci..

[57] Rengasamy K.R.R., Mahomoodally M.F., Aumeeruddy M.Z., Zengin G., Xiao J., Kim D.H. (2020). Bioactive Compounds in Seaweeds: An Overview of Their Biological Properties. Food Chem. Toxicol..

[58] Meinita M.D.N., Harwanto D., Sohn J.-H., Kim J.-S., Choi J.-S. (2021). *Hizikia fusiformis*: Pharmacological and Nutritional Properties. Foods.

[59] Rushdi M.I., Abdel-Rahman I.A.M., Saber H., Attia E.Z., Abdelraheem W.M., MAdkaur H.A., Hassan H.M., Elmaidomy H.A., Abdelmohsen U.R. (2020). Pharmacological and Natural Products Diversity of the Brown Algae Genus *Sargassum*. RSC Adv..

[60] Shobier A.H., El Ashry E.S. (2021). Pharmacological Applications of the Green Seaweed *Ulva lactuca*. Russ. J. Mar. Biol..

[61] Khotimchenko M., Tiasto V., Kalitnik A., Begum M., Khotimchenko R., Leonteva E., Bryukhovetskiy I., Khotimchenko Y. (2020). Antitumor Potential of Carrageenans from Marine Red Algae. Carbohydr. Polym..

[62] Sun H., Xu L., Wang K., Li Y., Bai T., Dong S., Wu H., Yao Z. (2023). κ-Carrageenan Oligosaccharides Protect Nerves by Regulating Microglial Autophagy in Alzheimer’s Disease. ACS Chem. Neurosci..

[63] Severino P., Da Silva C.F., Andrade L.N., De Lima Oliveira D., Campos J., Souto E.B. (2019). Alginate Nanoparticles for Drug Delivery and Targeting. Curr. Pharm. Des..

[64] Du Z.R., Wang X., Cao X., Liu X., Zhou S.N., Zhang H., Yang R.L., Wong K.H., Tang Q.J., Dong X.L. (2021). Alginate and its Two Components Acted Differently Against Dopaminergic Neuronal Loss in Parkinson’s Disease Mice Model. Mol. Nutr. Food Res..

[65] Apostolova E., Lukova P., Baldzhieva A., Katsarov P., Nikolova M., Iliev I., Peychev L., Trica B., Oancea F., Delattre C. (2020). Immunomodulatory and Anti-Inflammatory Effects of Fucoidan: A Review. Polymers.

[66] Maruyama H., Tamauchi H., Kawakami F., Yoshinaga K., Nakano T. (2015). Suppressive Effect of Dietary Fucoidan on Proinflammatory Immune Response and MMP-1 Expression in UVB-Irradiated Mouse Skin. Planta Med..

[67] Matsumoto S., Nagaoka M., Hara T., Kimura-Tagaki I., Mistuyama K., Ueyama S. (2004). Fucoidan derived from *Cladosiphon okamuranus* Tokida ameliorates murine chronic colitis through the down-regulation of interleukin-6 production on colonic epithelial cells. Clin. Exp. Immunol..

[68] Pozharitskaya O.N., Shikov A.N., Faustova N.M., Obluchinskaya E.D., Kosman V.M., Vuorela H., Makarov V.G. (2018). Pharmacokinetic and Tissue Distribution of Fucoidan from *Fucus vesiculosus* after Oral Administration to Rats. Mar. Drugs.

[69] Mamangam S., Krishnan D.A., Hillary V.E., Raja T.R.W., Mathew P., Kumar S.R., Paulraj M.G., Ignacimuthu S. (2020). Fucoidan Serves a Neuroprotective Effect in an Alzheimer’s Disease Model. Front. Biosci..

[70] Kumar L.R.G., Paul P.T., Anas K.K., Tejpal C.S., Chatterjee N.S., Anupama T.K., Mathew S., Ravishankar C.N. (2022). Phlorotannins-bioactivity and extraction perspectives. J. Appl. Phycol..

[71] Bogolitsyn K., Druzhinina A., Kaplitsin P., Ovchinnikov D., Parshina A., Kuznetsova M. (2019). Relationship between radical scavenging activity and polymolecular properties of brown algae polyphenols. Chem. Pap..

[72] Paudel P., Seong S.H., Wu S., Park S., Jung H.A., Choi J.S. (2019). Eckol as a Potential Therapeutic against Neurodegenerative Diseases Targeting Dopamine D₃/D₄ Receptors. Mar. Drugs.

[73] Barbosa M., Valentão P., Andrade P.B. (2020). Polyphenols from Brown Seaweeds (*Ochrophyta, Phaeophyceae*): Phlorotannins in the Pursuit of Natural Alternatives to Tackle Neurodegeneration. Mar. Drugs.

[74] Martins B., Vieira M., Delerue-Matos C., Grosso C., Soares C. (2022). Biological Potential, Gastrointestinal Digestion, Absorption, and Bioavailability of Algae-Derived Compounds with Neuroprotective Activity: A Comprehensive Review. Mar. Drugs.

[75] Xiang S., Liu F., Lin J., Chen H., Huang C., Chen L., Zhou Y., Ye L., Zhang K., Jin J. (2017). Fucoxanthin Inhibits β-Amyloid Assembly and Attenuates β-Amyloid Oligomer-Induced Cognitive Impairments. J. Agric. Food Chem..

[76] Meinita M.D.N., Harwanto D., Tirtawijaya G., Negara B.F.S.P., Sohn J.-H., Kim J.-S., Choi J.-S. (2021). Fucosterol of Marine Macroalgae: Bioactivity, Safety, and Toxicity on Organism. Mar. Drug.

[77] Wang P., Zhang J., Zhan N., Yang S., Yu M., Liu H. (2022). The pharmacokinetic characteristics and excretion studies of fucosterol from *Sargassum fusiforme* in rats. Biomed. Chromatogr..

[78] Bauer S., Jin W., Zhang F., Linhardt R.J. (2021). The Application of Seaweed Polysaccharides and Their Derived Products with Potential for the Treatment of Alzheimer’s Disease. Mar. Drugs.

[79] Alghazwi M., Charoensiddhi S., Smid S., Zhang W. (2020). Impact of *Ecklonia Radiata* Extracts on the Neuroprotective Activities against Amyloid Beta (Aβ1-42) Toxicity and Aggregation. J. Funct. Foods.

[80] Nordberg A., Ballard C., Bullock R., Darreh-Shori T., Somogyi M. (2013). A Review of Butyrylcholinesterase as a Therapeutic Target in the Treatment of Alzheimer’s Disease. Prim. Care Companion CNS Disord..

[81] Spehlmann R., Stahl S.M. (1976). Dopamine Acetylcholine Imbalance in Parkinson’s Disease: Possible Regenerative of Overgrowth of Cholinergic Axon Terminal. Lancet.

[82] Nho J.A., Shin Y.S., Jeong H.-R., Cho S., Heo H.J., Kim G.H., Kim D.-O. (2020). Neuroprotective Effects of Phlorotannin-Rich Extract from Brown Seaweed *Ecklonia Cava* on Neuronal PC-12 and SH-SY5Y Cells with Oxidative Stress. J. Microbiol. Biotechnol..

[83] Park S.K., Kang J.Y., Kim J.M., Park S.H., Kwon B.S., Kim G.-H., Heo H.J. (2018). Protective Effect of Fucoidan Extract from *Ecklonia Cava* on Hydrogen Peroxide-Induced Neurotoxicity. J. Microbiol. Biotechnol..

[84] Zorova L.D., Popkov V.A., Plotnikov E.Y., Silachev D.N., Pevzner I.B., Jankauskas S.S., Babenko V.A., Zorov S.D., Balakireva A.V., Juhaszova M. (2018). Mitochondrial Membrane Potential. Anal. Biochem..

[85] Silva J., Alves C., Pinteus S., Mendes S., Pedrosa R. (2018). Neuroprotective Effects of Seaweeds against 6-Hydroxidopamine-Induced Cell Death on an in Vitro Human Neuroblastoma Model. BMC Complement. Altern. Med..

[86] Silva J., Alves C., Pinteus S., Mendes S., Pedrosa R. (2020). Seaweeds’ neuroprotective potential set in vitro on a human cellular stress model. Mol. Cell Biochem..

[87] Huang C.-Y., Kuo C.-H., Chen P.-W. (2017). Compressional-Puffing Pretreatment Enhances Neuroprotective Effects of Fucoidans from the Brown Seaweed *Sargassum Hemiphyllum* on 6-Hydroxydopamine-Induced Apoptosis in SH-SY5Y Cells. Molecules.

[88] Olasehinde T.A., Olaniran A.O., Okoh A.I. (2019). Aqueous-Ethanol Extracts of Some South African Seaweeds Inhibit Beta-Amyloid Aggregation, Cholinesterases, and Beta-Secretase Activities in Vitro. J. Food Biochem..

[89] Yang W., Zhang Y., Li Y., Nie Y., Liang J., Liu Y., Liu J., Zhang Y., Song C., Qian Z. (2020). Chemical Composition and Anti-Alzheimer’s Disease-Related Activities of a Functional Oil from the Edible Seaweed *Hizikia Fusiforme*. Chem. Biodivers..

[90] Ko W., Lee H., Kim N., Jo H.G., Woo E.-R., Lee K., Han Y.S., Park S.R., Ahn G., Cheong S.H. (2021). The Anti-Oxidative and Anti-Neuroinflammatory Effects of *Sargassum horneri* by Heme Oxygenase-1 Induction in BV2 and HT22 Cells. Antioxidants.

[91] Fitton J., Dell’Acqua G., Gardiner V.-A., Karpiniec S., Stringer D., Davis E. (2015). Topical Benefits of Two Fucoidan-Rich Extracts from Marine Macroalgae. Cosmetics.

[92] Nishibori N., Sagara T., Hiroi T., Sawaguchi M., Itoh M., Her S., Mortika K. (2012). Protective effect of *Undaria pinnatifida* sporophyll extract on iron-induced cytotoxicity and oxidative stress in PC12 neuronal cells. Phytopharmacology.

[93] Caruana M., Camilleri A., Farrugia M.Y., Ghio S., Jakubíčková M., Cauchi R.J., Vassallo N. (2021). Extract from the Marine Seaweed *Padina Pavonica* Protects Mitochondrial Biomembranes from Damage by Amyloidogenic Peptides. Molecules.

[94] Shanmuganathan B., Sheeja Malar D., Sathya S., Pandima Devi K. (2015). Antiaggregation Potential of *Padina Gymnospora* against the Toxic Alzheimer’s Beta-Amyloid Peptide 25-35 and Cholinesterase Inhibitory Property of Its Bioactive Compounds. PLoS ONE.

[95] Machado L.P., Carvalho L.R., Young M.C.M., Cardoso-Lopes E.M., Centeno D.C., Zambotti-Villela L., Colepicolo P., Yokoya N.S. (2015). Evaluation of Acetylcholinesterase Inhibitory Activity of Brazilian Red Macroalgae Organic Extracts. Rev. Bras. Farmacogn..

[96] Sun M., Sheng Y., Zhu Y. (2021). Ginkolide B Alleviates the Inflammatory Response and Attenuates the Activation of LPS-Induced BV-2 Cells In Vitro and In Vivo. Exp. Ther. Med..

[97] Soares C., Paíga P., Marques M., Neto T., Carvalho A.P., Paiva A., Simões P., Costa L., Bernardo A., Fernández N. (2021). Multi-Step Subcritical Water Extracts of *Fucus vesiculosus* L. and *Codium tomentosum* Stackhouse: Composition, Health-Benefits and Safety. Processes.

[98] Milner J. (2009). Cellular Regulation of SIRT1. Curr. Pharm. Des..

[99] Herskovits A.J., Guarente L. (2014). SIRT1 in Neurodevelopment and Brain Senescence. Neuron.

[100] Godoy J.A., Zolezzi J.M., Braidy N., Inestrosa N.C. (2014). Role of SIRT1 during the Aging Process: Relevance to Protection of Synapses in the Brain. Mol. Neurobiol..

[101] Ghemrawi R., Pooya S., Lorentz S., Gauchotte G., Arnold C., Gueant J.L., Battaglia-Hsu S.-F. (2013). Decreased Vitamin B12 Availability Induces ER Stress Through Impaired SIRT1- Deacetylation of HSF1. Cell Death Dis..

[102] Li Y., Xu S., Giles A., Nakamura K., Lee J.W., Hou X., Donmez G., Li J., Lou Z., Walsh K. (2011). Hepatic Overexpression of SIRT1 in Mice Attenuates Endoplasmic Reticulum Stress and Insulin Resistance in the Liver. FASEB J..

[103] Jung T.W., Lee K.T., Lee M.W., Ka K.H. (2012). SIRT1 Attenuates Palmitate-Induced Endoplasmic Reticulum Stress and Insulin Resistance in HepG2 Cells Via Induction of Oxygen-Regulated Protein 150. Biochem. Biophys. Res. Commun..

[104] Tirtawijaya G., Mohibbullah M., Meinita M.D.N., Moon I.S., Hong Y.K. (2018). The Tropical Carrageenophyte Kappaphycus alvarezii Extract Promotes Axodendritic Maturation of Hippocampal Neurons in Primary Culture. J. Appl. Phycol..

[105] Tirtawijaya G., Mohibbullah M., Meinita M.D.N., Moon I.S., Hong Y.-K. (2016). The Ethanol Extract of the Rhodophyte *Kappaphycus alvarezii* Promotes Neurite Outgrowth in Hippocampal Neurons. J. Appl. Phycol..

[106] Lauretti E., Dincer O., Patrico D. (2020). Glycogen Synthase Kinase-3 Signaling in Alzheimer’s Disease. Biochim. Biophys. Acta Mol. Cell Res..

[107] Park S.K., Kang J.Y., Kim J.M., Yoo S.K., Han H.J., Chung D.H., Kim D.-O., Kim G.-H., Heo H.J. (2019). Fucoidan-Rich Substances from *Ecklonia Cava* Improve Trimethyltin-Induced Cognitive Dysfunction via Down-Regulation of Amyloid β Production/Tau Hyperphosphorylation. Mar. Drugs.

[108] Balendra V., Singh S.K. (2021). Therapeutic Potential of Astaxanthin and Super Oxide Dismutase in Alzheimer’s Disease. Open Biol..

[109] Haddad M., Herve V., Khedher M.R.B., Rabanel J.M., Ramassamy C. (2021). Glutathione: An Old and Small Molecule with Great Functions and New Applications in the Brain and in Alzheimer’s Disease. Antioxid. Redox Signal..

[110] Um M.Y., Lim D.W., Son H.J., Cho S., Lee C. (2018). Phlorotannin-Rich Fraction from *Ishige Foliacea* Brown Seaweed Prevents the Scopolamine-Induced Memory Impairment via Regulation of ERK-CREB-BDNF Pathway. J. Funct. Foods.

[111] Ye F., Wu A. (2021). The Protective Mechanism of SIRT1 in the Regulation of Mitochondrial Biogenesis and Mitochondrial autophagy in Alzheimer’s Disease. J. Alzheimer’s Dis..

[112] Gao L., Zhang Y., Sterling K., Song W. (2022). Brain-derived Neurotropic Factor in Alzheimer’s Disease and its Pharmaceutical Potential. Transl. Neurodegener..

[113] Liu X., Liu D., Lin G., Wu Y., Gao L., Ai C., Huang Y., Wang M., El-Seedi H.R., Chen X. (2019). Anti-Ageing and Antioxidant Effects of Sulfate Oligosaccharides from Green Algae Ulva Lactuca and Enteromorpha prolifera in SAMP8 Mice. Int. J. Biol. Macromol..

[114] Baek S.Y., Li F.Y., Kim D.H., Kim S.J., Kim M.R. (2020). Enteromorpha prolifera Extract Improves Memory in Scopolamine-Treated Mice via Downregulating Amyloid-β Expression and Upregulating BDNF/TrkB Pathway. Antioxidants.

[115] Briffa M., Ghio S., Neuner J., Gauci A.J., Cacciottolo R., Marchal C., Caruana M., Cullin C., Vassallo N., Cauchi R.J. (2017). Extracts from Two Ubiquitous Mediterranean Plants Ameliorate Cellular and Animal Models of Neurodegenerative Proteinopathies. Neurosci. Lett..

[116] Chudasama N.A., Sequeira R.A., Moradiya K., Prasad K. (2021). Seaweed Polysaccharide Based Products and Materials: An As-sessment on Their Production from a Sustainability Point of View. Molecules.

[117] Venkatesan J., Lowe B., Anil S., Manivasagan P., Al Kheraif A.A., Kang K.-H., Kim S.-K. (2015). Seaweed Polysaccharides and Their Potential Biomedical Applications. Starch Stärke.

[118] Pereira L. (2018). Biological and Therapeutic Properties of the Seaweed Polysaccharides. Int. Biol. Rev..

[119] Pereira L., Valado A. (2021). The Seaweed Diet in Prevention and Treatment of the Neurodegenerative Diseases. Mar. Drugs.

[120] Zhou R., Shi X.-Y., Bi D.-C., Fang W.-S., Wei G.-B., Xu X. (2015). Alginate-Derived Oligosaccharide Inhibits Neuroinflammation and Promotes Microglial Phagocytosis of β-Amyloid. Mar. Drugs.

[121] Souza R.B., Frota A.F., Silva J., Alves C., Neugebauer A.Z., Pinteus S., Rodrigues J.A.G., Cordeiro E.M.S., De Almeida R.R., Pedrosa R. (2018). In Vitro Activities of Kappa-Carrageenan Isolated from Red Marine Alga *Hypnea musciformis*: Antimicrobial, Anticancer and Neuroprotective Potential. Int. J. Biol. Macromol..

[122] Li Y., Zhang H., Kosturakis A.K., Cassidy R.M., Zhanh H., Kennamer-Chapman R.M., Jawad A.B., Colomand C.M., Harrison D.S., Dougherty P.M. (2015). MAPK Signaling Downstream to TLR4 Contributes to Paclitaxel-induced Peripheral Neuropathy. Brain Behav. Immun..

[123] Sakai J., Cammarota E., Wright J.A., Cicuta P., Gottschak R.A., Li N., Fraser I.D.C., Bryant C.E. (2017). Lipopolysaccharide-induced NF-κB nuclear translocation is primarily dependent on MyD88, but TNFα expression requires TRIF and MyD88. Sci. Rep..

[124] Kawai T., Akira S. (2007). Signaling to NF-kB by Toll Like Receptor. Trends Mol. Med..

[125] Kaminska B., Gozdz A., Zawazdka M., Ellert-Miklaszewska A., Lipko M. (2009). MAPK Signal Trunsduction Underlying Brain Inflammation and Gliosis as Therapeutic Target. Anat. Rec..

[126] Yao Z., Xu L., Jin L., Wang B., Fu C., Bai Y., Wu H. (2022). κ-Carrageenan Oligosaccharides Inhibit the Inflammation of Lipopolysaccharide-Activated Microglia Via TLR4/NF-ΚB and P38/JNK MAPKs Pathways. Neurochem. Res..

[127] Austin S., St-Pierre J. (2012). PGC1-α and Mitochondrial Metabolism-Enmerging Concept and Relevance in Ageing and Neurodegenerative Disorders. J. Cell Sci..

[128] Dinkova-Kostova A.T., Abramov A.Y. (2015). The Emerging Role of Nrf2 in Mitochondrial Fucntion. Free Radic. Biol. Med..

[129] Zhang L., Hao J., Zheng Y., Su R., Liao Y., Gong X., Liu L., Wang X. (2018). Fucoidan Protects Dopaminergic Neurons by Enhancing the Mitochondrial Function in a Rotenone-Induced Rat Model of Parkinson’s Disease. Aging Dis..

[130] Ramu S., Anbu J., Ammunje D.N., Krishnaraj K. (2022). Fucoidan Isolated from *Sargassum wightii* Greville Ameliorates Intracerebro-Ventricular Streptozotocin Induced Cognitive Deficits, Oxidative Stress and Amyloidosis in Wistar Rats. Bioact. Carbohydr. Diet. Fibre.

[131] Rocha C.P., Pacheco D., Cotas J., Marques J.C., Pereira L., Gonçalves A.M.M. (2021). Seaweeds as Valuable Sources of Essential Fatty Acids for Human Nutrition. Int. J. Environ. Res. Public Health.

[132] Lorenzo J., Agregán R., Munekata P., Franco D., Carballo J., Şahin S., Lacomba R., Barba F. (2017). Proximate Composition and Nutritional Value of Three Macroalgae: *Ascophyllum nodosum, Fucus vesiculosus* and *Bifurcaria bifurcata*. Mar. Drugs.

[133] Marques F., Lopes D., Da Costa E., Conde T., Rego A., Ribeiro A.I., Abreu M.H., Domingues M.R. (2021). Seaweed Blends as a Valuable Source of Polyunsaturated and Healthy Fats for Nutritional and Food Applications. Mar. Drugs.

[134] Susanto E., Fahmi A.S., Abe M., Hosokawa M., Miyashita K. (2016). Lipids, Fatty Acids, and Fucoxanthin Content from Temperate and Tropical Brown Seaweeds. Aquat. Procedia.

[135] Marinho G., Holdt S., Jacobsen C., Angelidaki I. (2015). Lipids and Composition of Fatty Acids of *Saccharina latissima* Cultivated Year-Round in Integrated Multi-Trophic Aquaculture. Mar. Drugs.

[136] Dyall S.C. (2015). Long-Chain Omega-3 Fatty Acids and the Brain: A Review of the Independent and Shared Effects of EPA, DPA and DHA. Front. Aging Neurosci..

[137] Bentsen H. (2017). Dietary Polyunsaturated Fatty Acids, Brain Function and Mental Health. Microb. Ecol. Health Dis..

[138] Beltz B.S., Tlusty M.F., Benton J.L., Sandeman D.C. (2007). Omega-3 Fatty Acids Upregulate Adult Neurogenesis. Neurosci. Lett..

[139] Qin L., He J., Hanes R.N., Pluzarev O., Hong J.-S., Crews F.T. (2008). Increased Systemic and Brain Cytokine Production and Neuroinflammation by Endotoxin Following Ethanol Treatment. J. Neuroinflamm..

[140] Hashimoto M., Hossain S., Tanabe Y., Kawashima A., Harada T., Yano T., Mizuguchi K., Shido O. (2009). The Protective Effect of Dietary Eicosapentaenoic Acid against Impairment of Spatial Cognition Learning Ability in Rats Infused with Amyloid β(1–40). J. Nutr. Biochem..

[141] Dyall S.C., Michael-Titus A.T. (2008). Neurological Benefits of Omega-3 Fatty Acids. Neuromol. Med..

[142] Dehkordi N.G., Noorbakhshnia M., Ghaedi K., Esmaeili A., Dabaghi M. (2015). Omega-3 Fatty Acids Prevent LPS-Induced Passive Avoidance Learning and Memory and *CaMKII-α* Gene Expression Impairments in Hippocampus of Rat. Pharmacol. Rep..

[143] De Andrade A.M., Da Cruz Fernandes M., De Fraga L.S., Porawski M., Giovenardi M., Guedes R.P. (2017). Omega-3 Fatty Acids Revert High-Fat Diet-Induced Neuroinflammation but Not Recognition Memory Impairment in Rats. Metab. Brain Dis..

[144] Mizushima N., Yoshimori T. (2007). How to Interpret LC3 Immunoblotting. Autophagy.

[145] Inoue T., Tanaka M., Masuda S., Ohue-Kitano R., Yamakage H., Muranaka K., Wada H., Kusakabe T., Shimatsu A., Hasegawa K. (2017). Omega-3 Polyunsaturated Fatty Acids Suppress the Inflammatory Responses of Lipopolysaccharide-Stimulated Mouse Microglia by Activating SIRT1 Pathways. Biochim. Biophys. Acta (BBA) Mol. Cell Biol. Lipids.

[146] Dong Y., Xu M., Kalueff A.V., Song C. (2018). Dietary Eicosapentaenoic Acid Normalizes Hippocampal Omega-3 and 6 Polyunsaturated Fatty Acid Profile, Attenuates Glial Activation and Regulates BDNF Function in a Rodent Model of Neuroinflammation Induced by Central Interleukin-1β Administration. Eur. J. Nutr..

[147] Dong Y., Pu K., Duan W., Chen H., Chen L., Wang Y. (2018). Involvement of Akt/CREB Signaling Pathways in the Protective Effect of EPA against Interleukin-1β-Induced Cytotoxicity and BDNF down-Regulation in Cultured Rat Hippocampal Neurons. BMC Neurosci..

[148] Barroso-Hernández A., Ramírez-Higuera A., Peña-Montes C., Cortés-Ramírez S.A., Rodríguez-Dorantes M., López-Franco Ó., Oliart-Ros R.M. (2022). Beneficial Effects of an Algal Oil Rich in ω-3 Polyunsaturated Fatty Acids on Locomotor Function and D2 Dopamine Receptor in Haloperidol-Induced Parkinsonism. Nutr. Neurosci..

[149] Taoro-González L., Pereda D., Valdés-Baizabal C., González-Gómez M., Pérez J.A., Mesa-Herrera F., Canerina-Amaro A., Pérez-González H., Rodríguez C., Díaz M. (2022). Effects of Dietary N-3 LCPUFA Supplementation on the Hippocampus of Aging Female Mice: Impact on Memory, Lipid Raft-Associated Glutamatergic Receptors and Neuroinflammation. Int. J. Mol. Sci..

[150] Luchtman D.W., Meng Q., Song C. (2012). Ethyl-Eicosapentaenoate (E-EPA) Attenuates Motor Impairments and Inflammation in the MPTP-Probenecid Mouse Model of Parkinson’s Disease. Behav. Brain Res..

[151] Lu D.-Y., Tsao Y.-Y., Leung Y.-M., Su K.-P. (2010). Docosahexaenoic Acid Suppresses Neuroinflammatory Responses and Induces Heme Oxygenase-1 Expression in BV-2 Microglia: Implications of Antidepressant Effects for Omega-3 Fatty Acids. Neuropsychopharmacology.

[152] Wu F., Wang D., Shi H., Wang C., Xue C., Wang Y., Zhang T. (2021). N-3 PUFA-Deficiency in Early Life Exhibits Aggravated MPTP-Induced Neurotoxicity in Old Age While Supplementation with DHA/EPA-Enriched Phospholipids Exerts a Neuroprotective Effect. Mol. Nutr. Food Res..

[153] Khan F., Jeong G.-J., Khan M.S.A., Tabassum N., Kim Y.-M. (2022). Seaweed-Derived Phlorotannins: A Review of Multiple Biological Roles and Action Mechanisms. Mar. Drugs.

[154] Lee S., Youn K., Kim D., Ahn M.-R., Yoon E., Kim O.-Y., Jun M. (2018). Anti-Neuroinflammatory Property of Phlorotannins from *Ecklonia cava* on Aβ_25–35_-Induced Damage in PC12 Cells. Mar. Drugs.

[155] Lee J., Jun M. (2019). Dual BACE1 and Cholinesterase Inhibitory Effects of Phlorotannins from *Ecklonia cava*—An In Vitro and in Silico Study. Mar. Drugs.

[156] Yoon J.-H., Lee N., Youn K., Jo M.R., Kim H.-R., Lee D.-S., Ho C.-T., Jun M. (2021). Dieckol Ameliorates Aβ Production via PI3K/Akt/GSK-3β Regulated APP Processing in SweAPP N2a Cell. Mar. Drugs.

[157] Peng J., Yuan J.-P., Wu C.-F., Wang J.-H. (2011). Fucoxanthin, a Marine Carotenoid Present in Brown Seaweeds and Diatoms: Metabolism and Bioactivities Relevant to Human Health. Mar. Drugs.

[158] Miyashita K., Beppu F., Hosokawa M., Liu X., Wang S. (2020). Nutraceutical Characteristics of the Brown Seaweed Carotenoid Fucoxanthin. Arch. Biochem. Biophys..

[159] Pangestuti R., Vo T.-S., Ngo D.-H., Kim S.-K. (2013). Fucoxanthin Ameliorates Inflammation and Oxidative Reponses in Microglia. J. Agric. Food Chem..

[160] Fernandes F., Barbosa M., Pereira D.M., Sousa-Pinto I., Valentão P., Azevedo I.C., Andrade P.B. (2018). Chemical Profiling of Edible Seaweed (*Ochrophyta*) Extracts and Assessment of Their in Vitro Effects on Cell-Free Enzyme Systems and on the Viability of Glutamate-Injured SH-SY5Y Cells. Food Chem. Toxicol..

[161] Yu J., Lin J.-J., Yu R., He S., Wang Q.-W., Cui W., Zhang J.-R. (2017). Fucoxanthin prevents H_2_O_2_-induced neuronal apoptosis via concurrently activating the PI3-K/Akt cascade and inhibiting the ERK pathway. Food Nutr. Res..

[162] Alghazwi M., Smid S., Musgrave I., Zhang W. (2019). In Vitro Studies of the Neuroprotective Activities of Astaxanthin and Fucoxanthin against Amyloid Beta (Aβ1-42) Toxicity and Aggregation. Neurochem. Int..

[163] Chu C.T., Plowey E.D., Dagda R.K., Hickey R.W., Cherra S.J., Clark R.S.B. (2009). Autophagy in Neurite Injury and Neurodegeneration. Methods Enzymol..

[164] Carnicella S., Drui G., Boulet S., Carcenac C., Favier M., Duran T., Savasta M. (2014). Implication of Dopamine D3 Receptor Activation in the Reversion of Parkinson Disease-Related Motivational Deficits. Transl. Psychiatry.

[165] Cho H.U., Kim S., Kim J., Yang S., An H., Nam M.H., Jang D.P., Lee C.J. (2021). Redefining Differential Role of MAO-A in Dopamine Degradation and MAO-B in Tonic GABA Synthesis. Exp. Mol. Med..

[166] Paudel P., Seong S.H., Jung H.A., Choi J.S. (2019). Characterizing Fucoxanthin as a Selective Dopamine D3/D4 Receptor Agonist: Relevance to Parkinson’s Disease. Chem. Biol. Interact..

[167] Sun G., Xin T., Zhang R., Liu C., Pang Q. (2020). Fucoxanthin Attenuates Behavior Deficits and Neuroinflammatory Response in 1-Methyl-4-Phenyl-1,2,3,6-Tetrahydropyridine-Induced Parkinson’s Disease in Mice. Pharmacogn. Mag..

[168] Kawasaki A., Ono A., Mizuta S., Kamiya M., Takenaga T., Murakami S. (2017). The Taurine Content of Japanese Seaweed. Adv. Exp. Med. Biol..

[169] Jakaria M., Azam S., Haque M.E., Jo S.-H., Uddin M.S., Kim I.-S., Choi D.-K. (2019). Taurine and Its Analogs in Neurological Disorders: Focus on Therapeutic Potential and Molecular Mechanisms. Redox Biol..

[170] Chung M., Malatesta P., Bosquesi P., Yamasaki P., Dos Santos J.L., Vizioli E. (2012). Advances in Drug Design Based on the Amino Acid Approach: Taurine Analogues for the Treatment of CNS Diseases. Pharmaceuticals.

[171] Oh S.J., Lee H.-J., Jeong Y.J., Nam K.R., Kang K.J., Han S.J., Lee K.C., Lee Y.J., Choi J.Y. (2020). Evaluation of the Neuroprotective Effect of Taurine in Alzheimer’s Disease Using Functional Molecular Imaging. Sci. Rep..

[172] Jang H., Lee S., Choi S.L., Kim H.Y., Baek S., Kim Y. (2017). Taurine Directly Binds to Oligomeric Amyloid-β and Recovers Cognitive Deficits in Alzheimer Model Mice. Adv. Exp. Med. Biol..

[173] Lu C.-L., Tang S., Meng Z.-J., He Y.-Y., Song L.-Y., Liu Y.-P., Ma N., Li X.-Y., Guo S.-C. (2014). Taurine Improves the Spatial Learning and Memory Ability Impaired by Sub-Chronic Manganese Exposure. J. Biomed. Sci..

[174] Kim H.Y., Kim H.V., Yoon J.H., Kang B.R., Cho S.M., Lee S., Kim J.Y., Kim J.W., Cho Y., Woo J. (2014). Taurine in Drinking Water Recovers Learning and Memory in the Adult APP/PS1 Mouse Model of Alzheimer’s Disease. Sci. Rep..

[175] Li X., Feng Y., Wang X.-X., Truong D., Wu Y.-C. (2020). The Critical Role of SIRT1 in Parkinson’s Disease: Mechanism and Therapeutic Considerations. Aging Dis..

[176] Terriente-Palacios C., Rubiño S., Hortós M., Peteiro C., Castellari M. (2022). Taurine, Homotaurine, GABA and Hydrophobic Amino Acids Content Influences “in Vitro” Antioxidant and SIRT1 Modulation Activities of Enzymatic Protein Hydrolysates from Algae. Sci. Rep..

[177] Tian T., Zhang B.Y., Wang K.D., Zhang B.F., Huang M. (2020). Protective Effects of Taurine on Neurons and Microglia in Parkinson’s Disease-like Mouse Model Induced by Paraquat. Zhonghua Lao Dong Wei Sheng Zhi Ye Bing Za Zhi.

[178] Wang K., Zhang B., Tian T., Zhang B., Shi G., Zhang C., Li G., Huang M. (2022). Taurine Protects Dopaminergic Neurons in Paraquat-Induced Parkinson’s Disease Mouse Model through PI3K/Akt Signaling Pathways. Amino Acids.

[179] Wang K., Shi Y., Liu W., Liu S., Sun M.-Z. (2021). Taurine Improves Neuron Injuries and Cognitive Impairment in a Mouse Parkinson’s Disease Model through Inhibition of Microglial Activation. Neurotoxicology.

[180] Che Y., Hou L., Sun F., Zhang C., Liu X., Piao F., Zhang D., Li H., Wang Q. (2018). Taurine Protects Dopaminergic Neurons in a Mouse Parkinson’s Disease Model through Inhibition of Microglial M1 Polarization. Cell Death Dis..

[181] Abuirmeileh A.N., Abuhamdah S.M., Ashraf A., Alzoubi K.H. (2021). Protective Effect of Caffeine and/or Taurine on the 6-Hydroxydopamine-Induced Rat Model of Parkinson’s Disease: Behavioral and Neurochemical Evidence. Restor. Neurol. Neurosci..

[182] Hou L., Che Y., Sun F., Wang Q. (2018). Taurine Protects Noradrenergic Locus Coeruleus Neurons in a Mouse Parkinson’s Disease Model by Inhibiting Microglial M1 Polarization. Amino Acids.

[183] Martens N., Schepers M., Zhan N., Leijten F., Voortman G., Tiane A., Rombaut B., Poisquet J., Sande N.v.d., Kerksiek A. (2021). 24(S)-Saringosterol Prevents Cognitive Decline in a Mouse Model for Alzheimer’s Disease. Mar. Drugs.

[184] Dai Y., Tan X., Wu W., Warner M., Gustafsson J.-Å. (2012). Liver X Receptor β Protects Dopaminergic Neurons in a Mouse Model of Parkinson Disease. Proc. Natl. Acad. Sci. USA.

[185] Chen Z., Liu J., Fu Z., Ye C., Zhang R., Song Y., Zhang Y., Li H., Ying H., Liu H. (2014). 24(*S*)-Saringosterol from Edible Marine Seaweed *Sargassum fusiforme* Is a Novel Selective LXRβ Agonist. J. Agric. Food Chem..

[186] Gan S.Y., Wong L.Z., Wong J.W., Tan E.L. (2019). Fucosterol Exerts Protection against Amyloid β-Induced Neurotoxicity, Reduces Intracellular Levels of Amyloid β and Enhances the MRNA Expression of Neuroglobin in Amyloid β-Induced SH-SY5Y Cells. Int. J. Biol. Macromol..

[187] Wong C.H., Gan S.Y., Tan S.C., Gany S.A., Ying T., Gray A.I., Igoli J., Chan E.W.L., Phang S.M. (2018). Fucosterol Inhibits the Cholinesterase Activities and Reduces the Release of Pro-Inflammatory Mediators in Lipopolysaccharide and Amyloid-Induced Microglial Cells. J. Appl. Phycol..

[188] Filippini M., Baldisserotto A., Menotta S., Fedrizzi G., Rubini S., Gigliotti D., Valpiani G., Buzzi R., Manfredini S., Vertuani S. (2021). Heavy Metals and Potential Risks in Edible Seaweed on the Market in Italy. Chemosphere.

[189] Chen Q., Pan X.-D., Huang B.-F., Han J.-L. (2018). Distribution of Metals and Metalloids in Dried Seaweeds and Health Risk to Population in Southeastern China. Sci. Rep..

[190] Ramu S., Murali A., Jayaraman A. (2019). Phytochemical Screening and Toxicological Evaluation of *Sargassum Wightii* Greville in Wistar Rats. Turk. J. Pharm. Sci..

[191] Tapia-Martínez J., Cano-Europa E., Casas-Valdez M., Blas-Valdivia V., Franco-Colin M. (2020). Toxicological and Therapeutic Evaluation of the Algae *Macrocystis pyrifera* (Phaeophyceae) in Rodents. Rev. Biol. Mar. Oceanogr..

[192] Taylor V.F., Li Z., Sayarath V., Palys T.J., Morse K.R., Scholz-Bright R.A., Karagas M.R. (2017). Distinct Arsenic Metabolites Following Seaweed Consumption in Humans. Sci. Rep..

[193] Lee D.H., Park M.Y., Shim B.J., Youn H.J., Hwang H.J., Shin H.C., Jeon H.K. (2012). Effects of *Ecklonia cava* Polyphenol in Individuals with Hypercholesterolemia: A Pilot Study. J. Med. Food.

[194] Shin H.-C., Kim S.H., Park Y., Lee B.H., Hwang H.J. (2012). Effects of 12-Week Oral Supplementation of *Ecklonia cava* Polyphenols on Anthropometric and Blood Lipid Parameters in Overweight Korean Individuals: A Double-Blind Randomized Clinical Trial. Phytother. Res..

[195] Choi E.-K., Park S.-H., Ha K.-C., Noh S.-O., Jung S.-J., Chae H.-J., Chae S.-W., Park T.-S. (2015). Clinical Trial of the Hypolipidemic Effects of a Brown Alga *Ecklonia cava* Extract in Patients with Hypercholesterolemia. Int. J. Pharmacol..

[196] Yun J.-W., Kim S.-H., Kim Y.-S., You J.-R., Cho E.-Y., Yoon J.-H., Kwon E., Yun I.-J., Oh J.-H., Jang J.-J. (2018). Enzymatic Extract from *Ecklonia cava*: Acute and Subchronic Oral Toxicity and Genotoxicity Studies. Regul. Toxicol. Pharmacol..

[197] Hwang P.-A., Yan M.-D., Lin H.-T., Li K.-L., Lin Y.-C. (2016). Toxicological Evaluation of Low Molecular Weight Fucoidan in Vitro and in Vivo. Mar. Drugs.

[198] Song M.Y., Ku S.K., Han J.S. (2012). Genotoxicity Testing of Low Molecular Weight Fucoidan from Brown Seaweeds. Food Chem. Toxicol..

[199] Bae M., Kim M.B., Park Y.K., Lee J.Y. (2020). Health benefits of fucoxanthin in the prevention of chronic diseases. Biochim. Biophys. Acta Mol. Cell Biol. Lipids.

[200] Turck D., Bresson J.-L., Burlingame B., Dean T., Fairweather-Tait S., Heinonen M., Hirsch-Ernst K.I., Mangelsdorf I., EFSA Panel on Dietetic Products, Nutrition and Allergies (NDA) (2017). Safety of *Ecklonia cava* phlorotannins as a novel food pursuant to Regulation (EC) No 258/97. EFSA J..

[201] Li Y., Qian Z.J., Kim M.M., Kim S.K. (2011). Cytotoxic activities of phlorethol and fucophlorethol derivatives isolated from Laminariaceae *Ecklonia cava*. J. Food Biochem..

[202] Kang M.C., Kang S.M., Ahn G., Kim K.N., Kang N., Samarakoon K.W., Oh M.C., Lee J.S., Jeon Y.J. (2013). Protective effect of a marine polyphenol, dieckol against carbon tetrachloride-induced acute liver damage in mouse. Environ. Toxicol. Pharmacol..

[203] Rajan D.K., Mohan K., Zhang S., Ganesan A.R. (2021). Dieckol: A brown algal phlorotannin with biological potential. Biomed. Pharmacother..

[204] Wang X., Sun G., Feng T., Zhang J., Huang X., Wang T., Xie Z., Chu X., Yang J., Wang H. (2019). Sodium Oligomannate Therapeutically Remodels Gut Microbiota and Suppresses Gut Bacterial Amino Acids-Shaped Neuroinflammation to Inhibit Alzheimer’s Disease Progression. Cell Res..

[205] Wang T., Kuang W., Chen W., Xu W., Zhang L., Li Y., Li H., Peng Y., Chen Y., Wang B. (2020). A Phase II Randomized Trial of Sodium Oligomannate in Alzheimer’s Dementia. Alzheimer’s Res. Ther..

[206] Xiao S., Chan P., Wang T., Hong Z., Wang S., Kuang W., He J., Pan X., Zhou Y., Ji Y. (2021). A 36-Week Multicenter, Randomized, Double-Blind, Placebo-Controlled, Parallel-Group, Phase 3 Clinical Trial of Sodium Oligomannate for Mild-to-Moderate Alzheimer’s Dementia. Alzheimer’s Res. Ther..

[207] Syed Y.Y. (2020). Sodium Oligomannate: First Approval. Drugs.

[208] Tolar M., Abushakra S., Hey J.A., Porsteinsson A., Sabbagh M. (2020). Aducanumab, gantenerumab, BAN2401, and ALZ-801-the first wave of amyloid-targeting drugs for Alzheimer’s disease with potential for near term approval. Alzheimer’s Res. Ther..

[209] Bolea I., Gella A., Unzeta M. (2013). Propargylamine-derived multitarget-directed ligands: Fighting Alzheimer’s disease with monoamine oxidase inhibitors. J. Neural Transm..

[210] Generalić Mekinić I., Skroza D., Šimat V., Hamed I., Čagalj M., Popović Perković Z. (2019). Phenolic Content of Brown Algae (*Pheophyceae*) Species: Extraction, Identification, and Quantification. Biomolecules.

[211] Wang S.-H., Huang C.-Y., Chen C.-Y., Chang C.-C., Huang C.-Y., Dong C.-D., Chang J.-S. (2021). Isolation and purification of brown algae fucoidan from *Sargassum siliquosum* and the analysis of anti-lipogenesis activity. Biochem. Eng. J..

[212] Maghraby Y.R., Farag M.A., Kontominas M.G., Shakour Z.T., Ramadan A.R. (2022). Nanoencapsulated Extract of a Red Seaweed (Rhodophyta) Species as a Promising Source of Natural Antioxidants. ACS Omega.

[213] Ramos-de-la-Peña A.M., Contreras-Esquivel J.C., Aguilar O., González-Valdez J. (2022). Structural and Bioactive Roles of Fucoidan in Nanogel Delivery Systems. A Review. Carbohydr. Polym. Technol. Appl..

[214] Zhang S., Qamar S.A., Junaid M., Munir B., Badar Q., Bilal M. (2022). Algal Polysaccharides-Based Nanoparticles for Targeted Drug Delivery Applications. Starch Stärke.

